# Serum Pharmacochemistry Combining Network Pharmacology to Discover the Active Constituents and Effect of Xijiao Dihuang Tang Prescription for Treatment of Blood-Heat and Blood-Stasis Syndrome-Related Disease

**DOI:** 10.1155/2022/6934812

**Published:** 2022-02-07

**Authors:** Yuxin Chen, Yang Dai, Jie Xia, Jing Liu, Guisheng Zhou, Cuihua Chen, Baoping Jiang, Lian Yin, Guochun Li

**Affiliations:** ^1^School of Pharmacy, Nanjing University of Chinese Medicine, 210046 Nanjing, China; ^2^School of Medicine & Holistic Integrative Medicine, Nanjing University of Chinese Medicine, 210046 Nanjing, China

## Abstract

Xijiao Dihuang Tang (XDT), a classic TCM prescription, has been used to clinically treat blood-heat and blood-stasis syndrome- (BHSS-) related diseases, including hemorrhagic stroke and sepsis. However, the active constituents and mechanism of XDT in the treatment of BHSS-related diseases have not been elucidated due to the lack of appropriate methodologies. In this study, serum pharmacochemistry and network pharmacology were used to explore the active constituents and the mechanism of XDT in the treatment of BHSS-related diseases. The effects of XDT were evaluated using dry yeast-induced rats as rat models with BHSS, which demonstrated the antipyretic and anticoagulant properties of XDT. The HPLC-QTOF/MS/MS assay was used to identify 60 serum constituents of XDT (SCXDT). Then, 338 targets of 60 SCXDT were predicted by integrating multiple databases and the MACCS fingerprint similarity prediction method. The degree of topological properties with targets of 19 key active constituents in SCXDT was identified and evaluated in glutamate-induced PC12 cells. Subsequently, 338 targets of 60 SCXDT were mainly involved in biological processes such as inflammation, coagulation, cell proliferation, and apoptosis, as well as oxidative contingencies via compound-target-disease network analysis. The core targets including IL-1*β*, IL-6, TNF, NOS3, and MAPK1 were identified using protein-protein interaction network analysis, whereas dozens of signaling pathways such as the p38MAPK signaling pathway were identified using functional pathway enrichment analysis. The results indicated that XDT has broad therapeutic and neuroprotective effects on inflammation, coagulation, oxidative stress, cell proliferation, and apoptosis in dry yeast-induced rats with BHSS and glutamate-induced PC12 cells by regulating the p38MAPK signaling pathway. This study not only discovered the active constituents of XDT but also elaborated its mechanisms in the treatment of BHSS-related diseases by intervening in a series of targets, signaling pathways, and biological processes such as inflammation, coagulation, oxidative stress, neuroprotection. The findings in this study provide a novel strategy for exploring the therapeutic efficacy of TCM prescriptions.

## 1. Introduction

Traditional Chinese medicine (TCM) has been clinically practiced for thousands of years with definite therapeutic effects and has multicomponent, multitarget, and multipath integrated treatment qualities. Because of the complexity of the chemical composition in TCM prescription and human body system, network pharmacology fits the multiconstituents, multitarget, and integrative treatment of diseases with TCM. Consequently, network pharmacology has become a common method to study the material basis and mechanism of TCM in recent years and has achieved promising preliminary results. However, TCM constituents are complex, and after metabolism, they are absorbed into the blood, where they must reach a certain blood concentration before they can be truly effective [1]. Serum pharmacochemistry strategies and methods greatly narrowed down the selection of active constituents of TCM. Therefore, in this paper, we adopted the serum pharmacochemistry combined with network pharmacology to analyze and identify the serum constituents of TCM, predict their acted targets and interactions, and systematically evaluate and validate the mechanisms of TCM extracts and serum containing *in vivo* and *in vitro* experiments. The purpose of this study was to provide new ideas and strategies for determining the pharmacological basis and mechanism of TCM.

Traditional Chinese Medicine syndrome is the basic concept of the TCM theory, which is a profile of symptoms and signs as a series of clinical phenotypes [2]. The blood-heat and blood-stasis syndrome (BHSS) is one of the basic TCM syndromes associated with a variety of various diseases, including hemorrhagic stroke and sepsis, with patients exhibiting elevated body temperature and abnormal blood rheological indexes [3]. Xijiao Dihuang Tang (XDT), which originated from Valuable Prescriptions for Emergency (Bei Ji Qian Jin Yao Fang) in China, is composed of four kinds of crude herbs including *Bubalus bubalis* Linnaeus (BB), *Rehmannia glutinosa* Libosch. (RG), *Paeonia lactiflora* Pall. (PL), and *Paeonia suffruticosa* Andr. (PS), and it can reduce C-reactive protein expression and improve coagulation status for the clinical treatment of sepsis with BHSS [4, 5]. Furthermore, network pharmacological prediction and experimental verification indicated that XDT improves sepsis survival by regulating the NF-*κ*B and HIF-1*α* signaling pathways [6, 7] and that it alleviates ischemic brain injury in MCAO rats by regulating inflammation, neurogenesis, and angiogenesis [8]. In addition, 71 compounds were identified through literature data mining and 237 XDT targets were predicted using a network pharmacology method, which involved signal transduction, transcriptional translocation, metabolic phase, apoptosis and proliferation, and immune process [9], but this has not been confirmed in experiments. There is a lack of systematic research on the active constituents and comprehensive therapeutic mechanism of XDT for the treatment of BHSS-related diseases.

**Figure 1:**
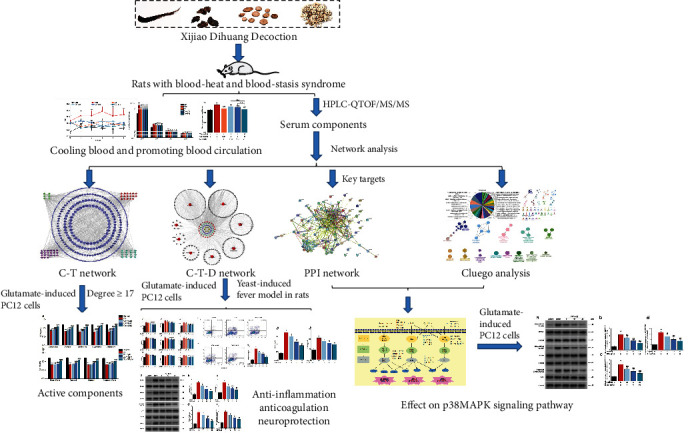
The overall production of XDT.

The purpose of this study was to identify the active constituents and reveal the mechanism of XDT for the treatment of BHSS-related diseases based on serum pharmacochemistry and network pharmacology. First, serum constituents of XDT (SCXDT) were identified using HPLC-QTOF/MS/MS methods, and network pharmacology was used to predict SCXDT targets, the disease network, the interaction of key targets, and signal pathways. Finally, the glutamate-induced PC12 cell model and yeast-induced fever rats with BHSS were used to verify the activity of the predicted main constituents, therapeutic effects, and mechanism of XDT. The overall procedure is illustrated in Figure 1.

## 2. Materials and Methods

### 2.1. Materials and Reagents

#### 2.1.1. Plant Material

*Bubalus bubalis* Linnaeus (20180820), *Rehmannia glutinosa* Libosch. (20181117), *Paeonia lactiflora* Pall. (20180201), and *Paeonia suffruticosa* Andr. (20190108) were purchased from Tong Ling Hetian Chinese Medicine Company (Tongling, China).

#### 2.1.2. Reference Samples

All drugs (purity assay by HPLC ≥ 98%, power) including constituents of geniposide, aucubin, salidroside, guanosine, adenosine, paeoniflorin, paeonol, catapol, and oxypaeoniflorin were supplied by the Chengdu Must Bio-Technology Co., Ltd. (Chengdu, China).

#### 2.1.3. Reagents

HPLC-grade ethanol was purchased from Merck Company (Darmstadt, Germany). Analytical grade acetic acid and ultrapure water (Watsons, Guangzhou, China) were used throughout the experiment. Instant dry yeast was obtained from Angel Yeast Co., Ltd. (Yichang, China). Aspirin enteric-coated tablets were supplied by Bayer S.p.A. (Viale Certosa, Milano, Italy). The cell counting kit 8 (CCK-8) was purchased from Nanjing Sunshine Biotechnology Co., Ltd. (Nanjing, China). The Annexin V-FITC/propidium iodide (PI) kit was supplied by Invitrogen (California, USA). Enzyme-linked Immunosorbent Assay (ELISA) Kits, Rat TNF-*α*, and Rat IL-1*β* were supplied by R&D Systems (Minnesota, USA). ELISA Kits for rat endothelin-1 (ET-1), rat intercellular adhesion molecule-1 (ICAM-1), rat matrix metallopeptidase 9 (MMP-9), rat NF-*κ*B, rat nitric oxide (NO), rat thromboxane B2 (TXB2), and rat superoxide dismutase (SOD) were procured from Nanjing Senbeijia Biological Technology Co., Ltd. (Nanjing, China).

### 2.2. Sample Preparation

XDT is composed of four kinds of crude drugs including *Bubalus bubalis* Linnaeus (BB), *Rehmannia glutinosa* Libosch. (RG), *Paeonia lactiflora* Pall. (PL), and *Paeonia suffruticosa* Andr. (PS). All XDT herbs were dried and smashed. The four drugs were mixed in the prescribed proportion to a total of 600 g. Initially, BB was boiled for two hours, and the solution was mixed with RG, PL, and PS before being boiled for 30 minutes. Next, water was added to the solution (1 : 8, *w*/*v*) before being boiled for 20 minutes. The double extraction solution was then concentrated under vacuum and dried, yielding 108 g XDT extracts from 600 g of the raw material. For animal experiments, 105.7 g of dried extract was dissolved in water to produce solutions with a concentration of 0.27 g/mL, 0.14 g/mL, and 0.07 g/mL. The solutions were stored at -20°C.

To produce solutions with a concentration of 0.18 g/g, 2.25 g of dried XDT extract was dissolved in 70% methanol. After centrifugation at 13, 000 × g for 10 minutes, 1 mL of the supernatant was filtered through a 0.45 *μ*m membrane filter, and 15 *μ*L filtrates were injected into the HPLC-QTOF/MS/MS.

Dry yeast and aspirin were dissolved in water at concentrations of 0.2 g/mL and 0.004 g/mL, respectively. Furthermore, geniposide, aucubin, salidroside, guanosine, adenosine, paeoniflorin, paeonol, catapol, and oxypaeoniflorin were dissolved in water at concentrations of 1 mmol/L.

### 2.3. Animals and Prescription Administration

Adult male Sprague-Dawley rats weighing 200–220 g were purchased from Shanghai JieSiJie Laboratory Animal Co., Ltd. (No. SCXK (Hu)2018-0004). All the animals were maintained under specific pathogen-free (SPF) conditions (22 ± 2°C, relative humidity of 50% ± 10%) under a 12-hour light, 12-hour dark cycle (lights turned on at 00:00 a.m.). All animal experiments were conducted in accordance with the guidelines of the Animal Experiments Committee and were approved by both the Science and Technology Department of Jiangsu Province as well as the Animal Care and Use Committee of the Nanjing University of Chinese Medicine.

Rats were randomly divided into six groups: the control, model, aspirin, and three XDT groups with varying concentrations (0.68 g/kg, 1.35 g/kg, and 2.70 g/kg). The 1.35 g/kg concentration was equivalent to the clinical dosage in adults. The treatment was administered orally once a day for seven days. Baseline rectal temperatures of the rats were taken using a digital thermometer before inducing fever. The depth of rectal measurement was 3 cm, and rats with rectal temperatures below 39°C (±5°C) were approved for use in the experiment. On the seventh day, fever was induced by subcutaneous injections of baker's yeast suspension (2 g/kg), followed by an 18-hour fast. Rectal temperatures of the rats were then taken at the 19^th^ hour, followed by administration of aspirin, XDT (0.68 g/kg, 1.35 g/kg, and 2.70 g/kg), and normal saline. The rectal temperatures of all rats were measured every hour after administration. Blood samples were collected from the rats five hours after administration.

Blood rheology indicators, including blood viscosity and plasma viscosity, were detected using an automatic blood rheology analyzer. Blood samples were placed in a water bath for 10 minutes at 37°C and centrifuged at 3500 rpm for 10 minutes to obtain the serum (supernatant), which was tested for the expression of ET-1, ICAM-1, TNF-*α*, IL-1*β*, MMP-9, NF-*κ*B, NO, SOD, and TXB2 using ELISA.

### 2.4. Analysis of Constituents

#### 2.4.1. Preparation of Serum Samples

Rats were randomly divided into two groups: model and XDT (1.35 g/kg). Treatments were administered orally twice daily for seven days and fasted with water for 12 hours before modeling. On the sixth day, rats in the XDT group were subcutaneously injected with a 20% yeast suspension (10 mL/kg) one hour after administration. On the seventh day, blood was collected one hour after administration. A solution of methanol (150 *μ*L) and serum (50 *μ*L) was formed and vortexed for approximately two minutes. The suspension was centrifuged at 1000 rpm for 10 minutes and dried using nitrogen gas at 35°C to obtain the supernatant. The residues were redissolved in 100 *μ*L methanol and centrifuged at 1000 rpm for 10 minutes. A 1 mL supernatant was subjected to HPLC-QTOF/MS/MS analysis. All samples were frozen at −80°C until the assay.

#### 2.4.2. HPLC-QTOF/MS/MS Analysis

For HPLC-QTOF/MS/MS analysis, an LC-20a Shimadzu HPLC system (Maryland, USA) was coupled to an orthogonal AB SCIEX Triple TOFTM 5600 mass spectrometry equipped with an electronic spray ionization (ESI) source. The chromatographic separation was performed on an ELITE C_18_ ODS HYPERSIL column (4.6 mm × 250 mm, 5 *μ*m) at 25°C. A mixture of solvent A (ethanol) and solvent C (0.1% acetic acid, *v*/*v*) was used as the mobile phase at a flow rate of 0.5 mL/min. The gradient elution program was as follows: 0–5 minutes, 4–30% A; 5–10 minutes, 30-35% A; 10–15 minutes, 35–45% A; 15–25 minutes, 45–45% A; 25–30 minutes, 45–50% A; 30–40 minutes, 50-60% A; and 40–50 minutes, 60–100% A. This was followed by a 15-minute equilibrium period before injecting the next sample at an injection volume of 15 *μ*L. For the full-scan MS analysis, ESI-MS spectra were acquired in both positive and negative ion modes, with negative ion mode performing better, and the spectra were recorded in the range of *m*/*z* 50–1500. The conditions of MS analysis were designed as follows: capillary voltage, 2800 V; the source temperature, 100°C; the cone voltage, 20 V; data collected mode, dynamic background deduction and information-dependent acquisition; MCR detection voltage, 2100 V; collision energy, 10 V; spray voltage, 20 V; nebulizer, 55 psi; aux gas pressure, 60 psi; curtain gas, 40 psi; IS, 20 V, gas flow, 450 (L/HR); desolvation temp, 250°C; injection volume, 15 *μ*L; detector, time of flight mass spectrometer; and delisting potential, −70 V.

### 2.5. Cellular Experiments

#### 2.5.1. Preparation for Containing Serum of XDT (XDT-cs)

Five rats were given 1.35 g/kg XDT extracts orally at once a day for five days. Blood samples were obtained from the rats one hour after the last administration. The blood samples were placed in a water bath for 10 minutes at 37°C before being centrifuged at 3500 rpm for 10 minutes to obtain the serum (supernatant).

#### 2.5.2. Cell Culture and Treatment

PC12 cells were purchased from American Tissue Culture Collection (ATCC, Manassas, VA, USA) and cultured at 37°C in Dulbecco's modified Eagle medium (DMEM) containing 10% (*v*/*v*) heat-inactivated fetal bovine serum (FBS, Hyclone), 100 U/mL penicillin, and 100 *μ*g/mL streptomycin (Sigma-Aldrich, MO). When cells were ~90% confluent, a conditioned medium was collected and centrifuged at 1000 rpm for five minutes to remove supernatant cells. The cells were added to the culture medium and resuscitated, and the remaining adherent cells were washed twice with 2 mL of phosphate-buffered saline (PBS). After discarding the PBS, 2 mL 0.25% (*w*/*v*) Trypsin-0.53 mM ethylenediaminetetraacetic acid (EDTA) mixed digest was added and observed under a microscope for about one minute. After the cells had been rounded, 2 mL of complete medium was quickly added to stop digestion and gently pipetted to collect the cells. The supernatant was discarded after centrifugation at 1000 rpm for five minutes, and the cells were resuspended in complete medium and mixed with the suspension cells (changed every other day), cultured in divided bottles.

#### 2.5.3. Cell Seeding Plate and Induction

Cells were dissociated with 0.25% trypsin and 0.5 mM EDTA (Gibco BRL) at 37°C for one minute and collected by centrifugation at 1000 rpm for five minutes. After the supernatant was discarded, a fresh medium was added and the cells were evenly blown. Cell suspension (20 *μ*L) was mixed with 4% trypan blue solution (20 *μ*L). When the cells were more than ~90% confluent, they were counted to adjust the density to 1 × 10^5^/mL. The cell suspension was plated into six well plates at 2 mL per well and incubated in a cell incubator at 37°C with 5% CO_2_ for 24 hours. For differentiation, the culture medium was replaced with DMEM supplemented with 1% FBS, 1% penicillin/streptomycin, and 50 ng/mL nerve growth factor (NGF) (Sigma-Aldrich, USA). The cells were collected after 48 hours of induction, and their concentration was adjusted to 5000 cells/100 *μ*L. In each well of 96-well plates, 100 *μ*L cell suspension was seeded and incubated overnight at 37°C and 5% CO_2_.

#### 2.5.4. Cell Counting Kit-8 Assay

Cell viability was analyzed using the cell counting kit-8 (CCK-8) assay. Cells were seeded in 96-well plates at a density of 5000 cells/well. After incubation with the germ-free serums (5%XDT-cs, 10% XDT-cs, and 20% XDT-cs) or drug solution (geniposide, aucubin, salidroside, guanosine, adenosine, paeoniflorin, paeonol, catapol, and oxypaeoniflorin) cotreated with glutamate for the indicated period, 10 *μ*L CCK-8 was added to each well. The 96-well plates were maintained at 37°C for four hours. The absorbance (OD value) was measured at a wavelength of 450 nm with enzyme-linked immunity implemented. Experiments in each group were performed in triplicate.

#### 2.5.5. Flow Cytometry (FCM) with PI Staining

The cells were cotreated with glutamate for the indicated time after incubation with the germ-free serums (5% XDT-cs, 10% XDT-cs, and 20% XDT-cs). Following the manufacturer's instructions, 1 × 10^6^ cells/1 mL were harvested by centrifugation at 1000 rpm for five minutes, washed in cold PBS at 4°C, and resuspended in 1 *μ*L FITC-labeled Annexin V. Next, 1 *μ*L PI (100 *μ*g/mL) and 5 *μ*L Alexa Fluor 488 Annexin V were added to each 100 *μ*L cell suspension. The flow cytometer was used to analyze cell apoptosis after 15 minutes of incubation at room temperature in the dark. Experiments in each group were performed in triplicate.

#### 2.5.6. Enzyme-Linked Immunosorbent Assay

After incubation with 5%XDT‐cs + glutamate, 10%XDT‐cs + glutamate, 20%XDT‐cs + glutamate, glutamate, and control groups, PC12 cell supernatants were collected after centrifugation at 800 rpm and tested for the presence of TNF-*α* and IL-1*β* using ELISA.

#### 2.5.7. Western Blot (WB) Analysis

Cells were lysed in radioimmunoprecipitation assay (RIPA) buffer (Beyotime, Shanghai, China) containing 1% phenylmethylsulfonyl fluoride (PMSF), followed by centrifugation at 12,000 rpm for five minutes at 4°C. The protein concentration was determined using BCA Protein Assay Kit. The supernatant was mixed with a sample loading buffer containing 5 × sodium dodecyl sulfate-polyacrylamide gel electrophoresis (SDS-PAGE), and an equal amount of protein was separated by SDS-8%-4% PAGE and transferred to a polyvinylidene fluoride (PVDF) membrane (Millipore, Schwalbach, Germany). The membrane was then blocked with 5% nonfat milk in TBS with Tween-20 (TBST) at room temperature for one hour, before being incubated overnight at 4°C with antibodies against phosphomitogen-activated protein kinase kinase 3 (Phos-MKK-3) (1 : 1000, Abcam, Cambridge, UK, catalogue number: ab131283), MKK-3 (1 : 1000, CST, Boston, USA, catalogue number: 8535), Phos-MKK-6 (1 : 500, BIOTECHNOLOGY, CA, USA, catalogue number: A7154), MKK-6 (1 : 1000, CST, catalogue number: 9264), Phos-p38 (1 : 1000, CST, catalogue number: 4511), P38 (1 : 5000, CST, catalogue number: 8690), ICAM-1 (1 : 5000, Abcam, catalogue number: ab206398), MMP-9 (1 : 20 000, Abcam, ab76003), Phos-p65 (1 : 1000, CST, catalogue number: 3033), P65 (1 : 1000, CST, catalogue number: 8242), COX-2 (1 : 1000, CST, catalogue number: 12282), and *β*-actin (1 : 5000). The membrane was washed and then incubated with 5% nonfat milk in TBST containing goat anti-rabbit immunoglobulin G-horseradish peroxidase (IgG-HRP) (Abcam, catalog number: ab205718) or goat anti-mouse IgG- HRP (Abcam, catalog number: ab205719) at room temperature for one hour. Protein bands were visualized using a chemiluminescence (ECL) solution (Thermo Fisher Scientific) and detected using a Tanon 6600 Luminescence Imaging Workstation (Tanon, China).

### 2.6. Network Pharmacological Analysis of SCXDT

#### 2.6.1. Target Prediction

The SCXDT identified using HPLC-Q/TOF-MS/MS analysis were considered candidate constituents. The potential molecular targets of prototype constituents in XDT-cs were predicted using Swiss Target Prediction (STP) [10] and the TCM for Systems Pharmacology Database and Analysis Platform (TCMSP) [11]. The Tanimoto coefficient was used to define the similarity score (score ≥ 0.4), and the potential targets were obtained based on the multiply-accumulate operations (MACCS) key fingerprint of the parent nuclear structure of its metabolic constituents in XDT-cs and the two-dimensional (2D) molecular structure of related drugs collected from drug database or inhibitors of related signaling pathways.

#### 2.6.2. Compound-Target Network (C-T Network)

The C-T network was constructed and visualized using Cytoscape version 3.7.1 [12] based on the compound-target interaction obtained in the previous step.

#### 2.6.3. Compound-Target-Disease Network (C-T-D Network)

A C-T-D network was built using network visualization software Cytoscape 3.7.1 based on SCXDT, targets, and related diseases.

#### 2.6.4. Pathway Analysis

Pathway analysis with GlueGo [12] was performed on 338 targets actuated by SCXDT to analyze biological interpretation and interrelationships of functional targets in biological networks.

#### 2.6.5. Protein-Protein Interaction Networks (PPI)

The targets derived from inner circle 1 and inner circle 2 in the C-T network diagram were the core target of SCXDT. The STRING online database was used to obtain the PPI data of the core targets of SCXDT [13], with the parameter organism set to *Homo sapiens* and the other basic settings left at the default value. Cytoscape software was used to establish the PPI relationship network and perform topological analysis.

### 2.7. Statistical Analysis

All results are expressed as the mean ± standard deviation (SD). Data were analyzed using SPSS 18.0 software (SPSS, Inc., Chicago, IL, USA) and GraphPad Prism (version 8.0, GraphPad Software Inc., San Diego, CA, USA). Differences between three or more groups at one point were analyzed using one-way analysis of variance (ANOVA). *P* values < 0.05 were considered statistically significant.

## 3. Results

### 3.1. XDT Has Antipyretic and Anticoagulant Effects on Yeast-Induced Fever Rats

**Figure 2:**
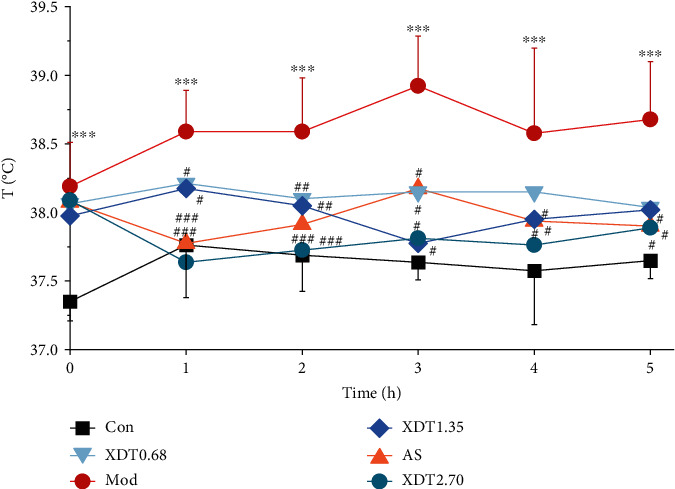
The rectal temperature of XDT and AS on yeast-induced fever rats. The rectal temperature of yeast-induced fever rats (Mod) and rats treated with 0.9% saline (Con), aspirin (AS), 0.68 g/kg XDT (XDT0.68), 1.35 g/kg XDT (XDT1.35), and 2.70 g/kg XDT (XDT2.70). The values represent the mean ± SD. ^∗∗^*P* < 0.01 and^∗∗∗^*P* < 0.001 vs. the control group; ^#^*P* < 0.05,  ^##^*P* < 0.01, and^###^*P* < 0.001 vs. the model group; *n* = 8.

The antipyretic and anticoagulant effects of XDT on yeast-induced fever rats were evaluated. The rectal temperature of the rats in each group was recorded within five hours after intragastric administration. Rats in the model group had significantly higher rectal temperatures (*P* < 0.001 vs. control group) (Figure 2). The rectal temperature of rats in the aspirin (AS) and the XDT groups was suppressed at 2.70 g/kg, 1.35 g/kg, and 0.68 g/kg (*P* < 0.001, *P* < 0.01, or *P* < 0.05 vs. the model group). It was found that XDT had an antipyretic effect on yeast-induced fever rats. Furthermore, XDT at 2.70 g/kg had a better antipyretic effect than aspirin after five hours (*P* < 0.001 or *P* < 0.05 vs. the model group).

**Figure 3:**
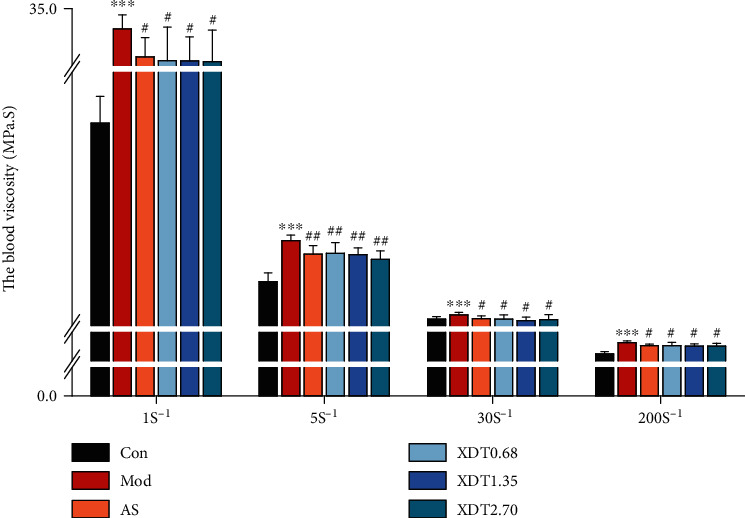
The blood viscosity of XDT and AS on yeast-induced fever rats. The blood viscosity of yeast-induced fever rats (Mod) and rats treated with 0.9% saline (Con), aspirin (AS), 0.68 g/kg XDT (XDT0.68), 1.35 g/kg XDT (XDT1.35), and 2.70 g/kg XDT (XDT2.70). The values represent the mean ± SD. ^∗∗∗^*P* < 0.001 vs. the control group; ^#^*P* < 0.05,  ^##^*P* < 0.01, and^###^*P* < 0.001 vs. the model group; *n* = 8.

The blood viscosity of rats in the model group increased significantly (*P* < 0.001) compared to the control group (Figure 3). When compared to the model group, the blood viscosity of rats in the AS group and XDT at 0.68 g/kg, 1.35 g/kg, and 2.70 g/kg at high, medium, and low shear rate was significantly reduced (*P* < 0.05 or *P* < 0.01).

**Figure 4:**
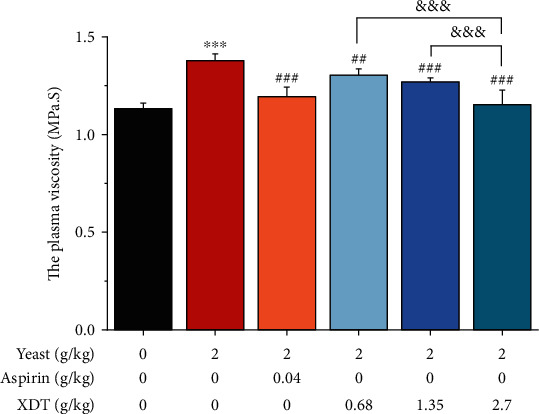
The plasma viscosity of XDT and AS on yeast-induced fever rats. The plasma viscosity of yeast-induced fever rats (Mod) and rats treated with 0.9% saline (Con), aspirin (AS), 0.68 g/kg XDT (XDT0.68), 1.35 g/kg XDT (XDT1.35), and 2.70 g/kg XDT (XDT2.70) after administration. The values represent the mean ± SD. ^∗∗∗^*P* < 0.001 vs. the control group; ^#^*P* < 0.05,  ^##^*P* < 0.01, and^###^*P* < 0.001 vs. the model group; ^&&&^*P* < 0.001 vs. the XDT2.70 group; *n* = 8.

The plasma viscosity of rats in the model group increased significantly (*P* < 0.001 vs. model group) compared to the control group (Figure 4). When compared to the model group, the plasma viscosity of rats in AS and XDT at 0.68 g/kg, 1.35 g/kg, and 2.70 g/kg was significantly reduced (*P* < 0.05, *P* < 0.01, or *P* < 0.001), demonstrating the anticoagulant activity of AS and XDT. Furthermore, XDT inhibited plasma viscosity in a dose-dependent manner.

### 3.2. Serum Constituents of XDT Analysis

**Figure 5:**
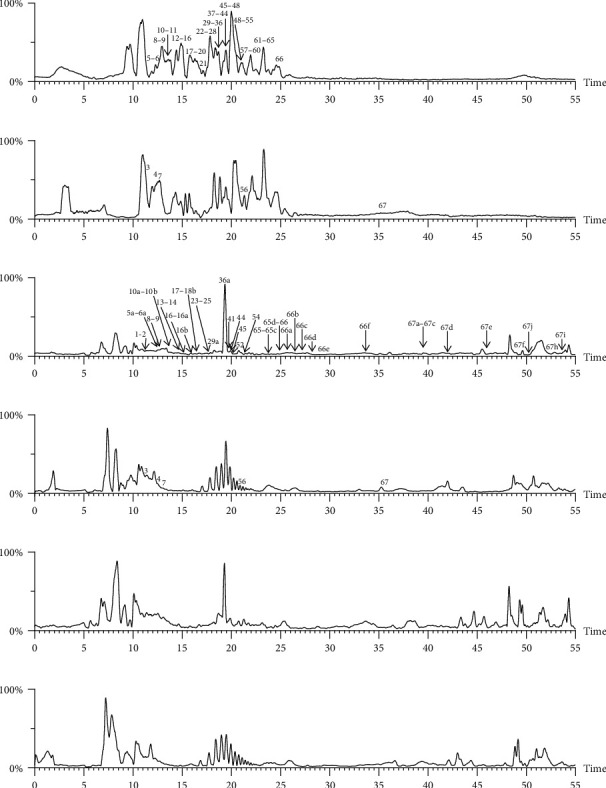
The total ion chromatograms (TIC) of XDT extract in negative mode (a) and positive mode (b), containing serum of XDT (XDT-cs) in negative mode (c) and positive mode (d), and serum samples from rats in negative mode (e) and positive mode on yeast-induced fever rats (f).

To accurately analyze SCXDT, the HPLC-QTOF/MS/MS method was used to compare the total ion current chromatograms (TIC) of XDT extracts and XDT-cs in the positive and negative ion modes (Figure 5). A total of 67 peaks were detected in the TIC of XDT extracts and 60 peaks in TIC of XDT-cs (Table S1). Twelve standard available constituents (constituents 13, 16, 18, 22, 24, 25, 41, 42, 43, 45, 56, and 65) were, respectively, identified as guanosine, catalpol, geniposidic acid, catechin, salidroside, oxypaeoniflorin, geniposide, genipin, albiflorin, paeoniflorin, adenosine, and paeonol by comparing sample retention times and accurate masses with those of the standards. For the standard unavailable constituents, a series of continuous procedures was used to increase the credibility for structure identification by calculating the molecular formulas based on high-precision quasimolecular ions such as [M-H] ^–^ and [M+H] ^+^ with a mass error of 5.0 ppm. Furthermore, the MS^n^ information was used to confirm the structure of the constituents by comparing the fragmentation paths to 12 standards or related literature.

Overall, 23 prototype and 37 metabolic constituents were initially identified in SCXDT, including 26 monoterpenoids, 11 iridoid glucosides, one flavonoid, nine phenols, four amino acids, two nucleosides, and seven phenylethanoid glycosides. The report revealed that there are four main amino acids in BB, one main monoterpenoid and 11 iridoid glucosides in RG, 14 main monoterpenoids in PL, and 11 main monoterpenoids in PS in SCXDT.

### 3.3. Prediction and Verification of Active Constituents in Serum Constituents of XDT

#### 3.3.1. C-T Network in Serum Constituents of XDT

**Figure 6:**
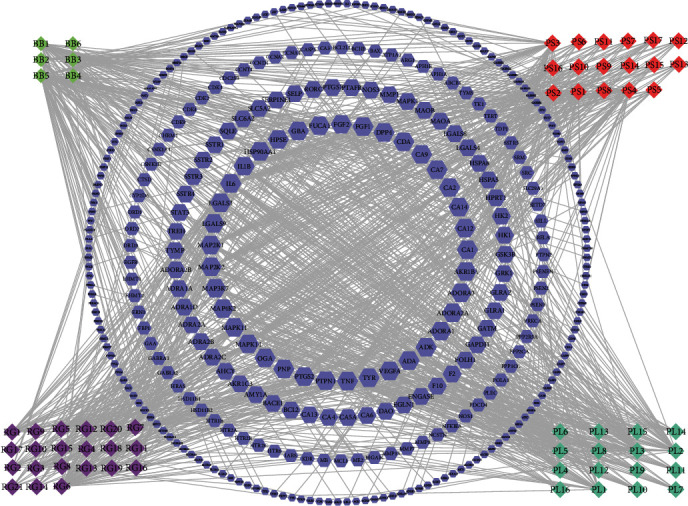
C-T network of SCXDT. The figure was constructed by combining 60 SCXDT and 338 corresponding targets. The diamond represents constituents. Each color represents a single drug. The blue hexagon represents the target. Targets in the inner circle with larger sizes display more relationships with constituents than those in the outer circles. BB: *Bubalus bubalis* Linnaeus; RG: *Rehmannia glutinosa* Libosch.; PL: *Paeonia lactiflora* Pall.; PS: *Paeonia suffruticosa* Andr.

Because TCM has the effect of a multicomponent comprehensive treatment, the potential pharmacological mechanism of XDT was studied using network pharmacology. Initially, 338 targets of 60 SCXDT were mined using the TCMSP, STP database, and MACCS fingerprint similarity prediction method (Table S2). Second, the C-T network was constructed using Cytoscape based on SCXDT and predicted targets (Figure 6). The network had a total of 398 nodes, 60 constituent nodes, and 338 target nodes, with 985 constituent-target linkages demonstrating that XDT has a comprehensive effect on multiple constituents and targets. The degree of topological properties was used to filter 19 constituents with values greater than or equal to 17 connected between SCXDT and the targets (Table 1), which were considered to be the core constituents of SCXDT.

#### 3.3.2. Effects of Core Constituents in SCXDT on Glutamate-Induced PC12 Cells

**Figure 7:**
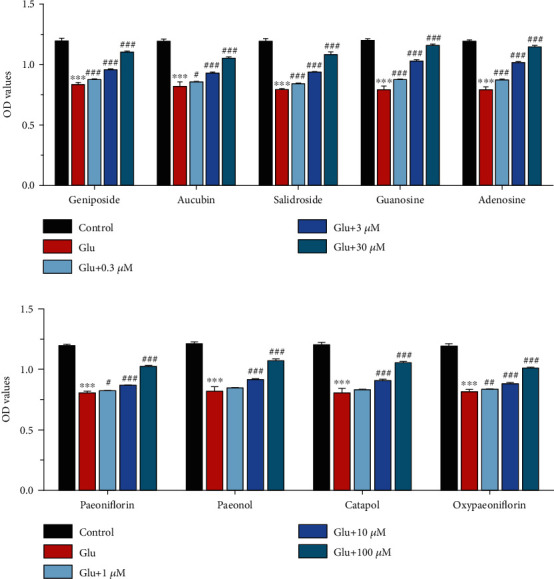
Effect of core constituents in SCXDT on OD values by CCK-8 assay in response to glutamate-induced PC12 cells. (a) Geniposide, aucubin, salidroside, guanosine, and adenosine groups; (b) paeoniflorin, paeonol, catapol, and oxypaeoniflorin groups. Data are expressed as the mean ± SD. ^∗∗∗^*P* < 0.001 vs. the control group; ^#^*P* < 0.05,  ^##^*P* < 0.01, and^###^*P* < 0.001 vs. the model group; *n* = 3.

The activity of the predicted core constituents of SCXDT was measured in glutamate-induced PC12 cells using CCK-8 assay. The results demonstrated that the viability of the model group was significantly lower than that of the control group (*P* < 0.001), and the viability of the geniposide, aucubin, salidroside, guanosine, adenosine, paeoniflorin, paeonol, catapol, and oxypaeoniflorin groups was significantly higher than that of the model group (*P* < 0.05 or *P* < 0.001, Figure 7). It was hypothesized that the core constituents of SCXDT can increase cell proliferation in glutamate-induced PC12 cells while also providing neuroprotection.

### 3.4. Prediction and Verification of the Comprehensive Effect of XDT

#### 3.4.1. C-T-D Network in Serum Constituents of XDT

**Figure 8:**
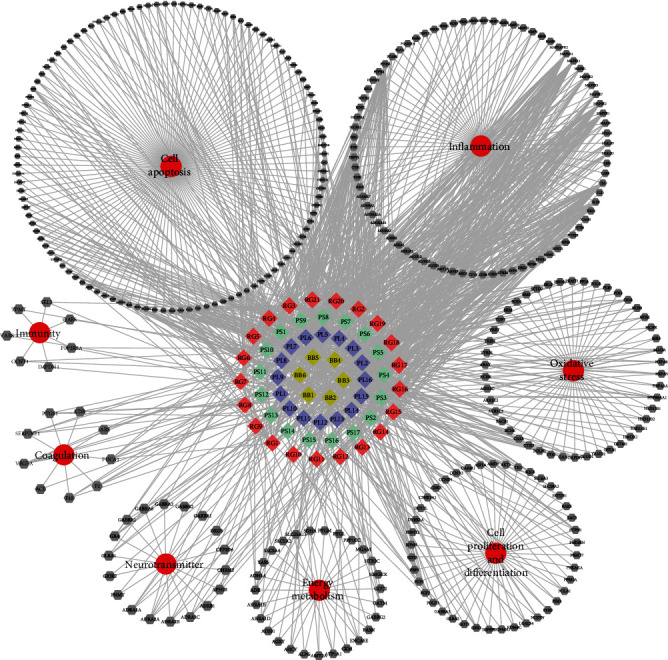
C-T-D network in SCXDT. There were 60 constituents, 338 corresponding targets, and 8 corresponding pathological types in the C-T-D network. The diamond represents constituents, each color represents one drug in XDT, respectively, and grey hexagon represents targets while the red circles represent the pathological types. BB: *Bubalus bubalis* Linnaeus; RG: *Rehmannia glutinosa* Libosch.; PL: *Paeonia lactiflora* Pall.; PS: *Paeonia suffruticosa* Andr.

The C-T-D network of SCXDT was constructed to predict and interpret the polypharmacology action of multicompound and multitarget to further reveal the multichannel comprehensive treatment effect of XDT on the treatment of BHSS-related disease. There were 60 SCXDT, 338 corresponding targets, and eight main pathologies with the connections totaling 1323 items, indicating their close relationships (Figure 8). Many of the targets were independently associated with cell apoptosis (111/338), inflammation (79/338), oxidative stress (46/338), cell proliferation and differentiation (41/338), energy metabolism (27/338), neurotransmitter (18/338), coagulation (9/338), and immunity (7/338), implying that the SCXDT may intervene in many biological processes in the treatment of BHSS-related diseases. For example, TNF (TNF-*α*), IL-6, and IL-1*β*, which are inflammatory factors, had a high degree of connectivity and played an important role in the inflammatory response. Furthermore, F2 and F10 are coagulation factors that affect blood coagulation. If prostaglandin-endoperoxide synthase 2 (PTGS2) is deleted in mice, the expression of TF through Annexin A2 (ANXA2) is increased. The exogenous coagulation pathway, which also leads to a hypercoagulable state, is initiated by TF [14].

#### 3.4.2. Effect of XDT on Yeast-Induced Fever in Rats

**Figure 9:**
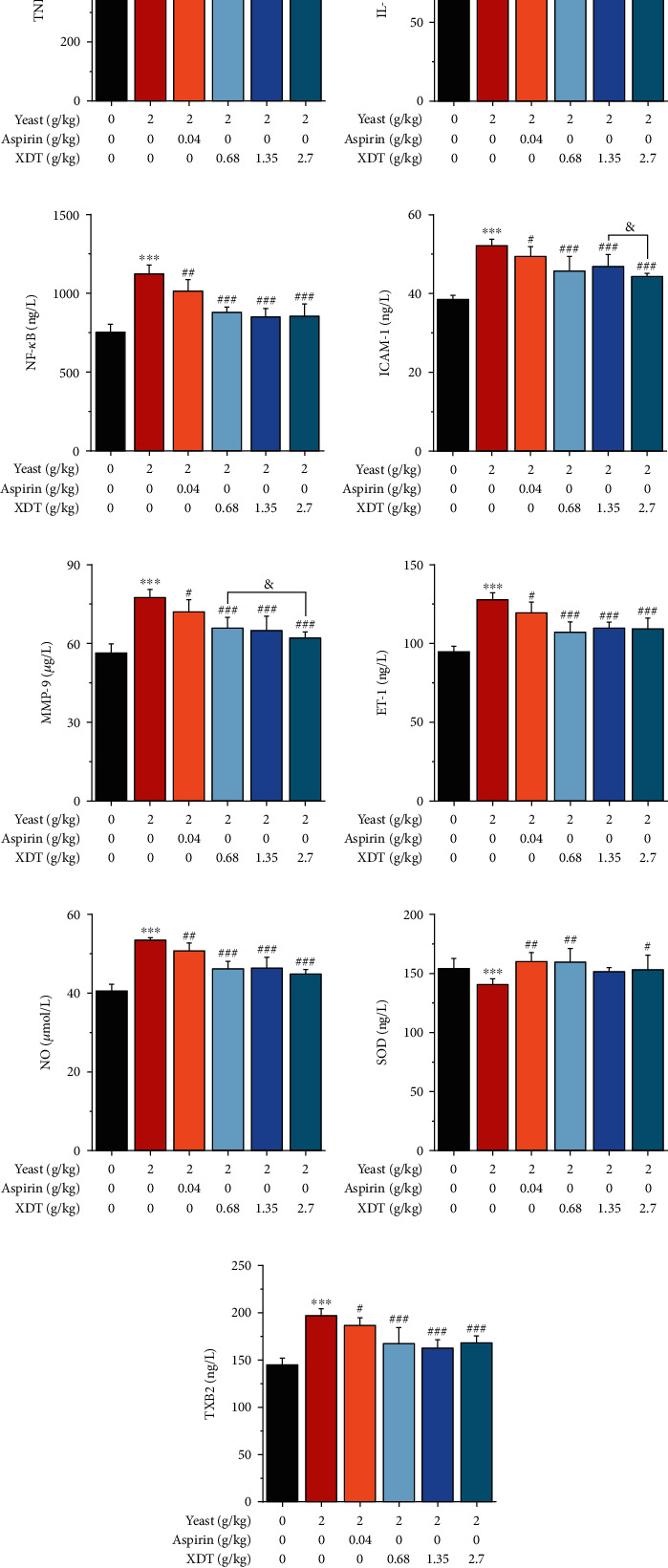
Effect of XDT on the level of TNF-*α*, IL-1*β*, NF-*κ*b, ICAM-1, MMP-9, ET-1, NO, SOD, and TXB2 on yeast-induced fever in rats by ELISA. Data are expressed as the mean ± SD. ^∗∗∗^*P* < 0.001 vs. the control group; ^#^*P* < 0.05,  ^##^*P* < 0.01, and^###^*P* < 0.001 vs. the model group; ^&^*P* < 0.05 vs. the XDT 2.70 group; ^¥^*P* < 0.05 vs. the XDT 0.68 group; *n* = 8.

The anti-inflammatory, anticoagulant, and antioxidant activities of XDT in the yeast-induced fever model were evaluated using ELISA based on the prediction on pharmacology action by C-T-D network analysis (Figure 8). The results demonstrated that the levels of ET-1, ICAM-1, TNF-*α*, IL-1*β*, MMP-9, NF-*κ*B, NO, and TXB2 in yeast-induced fever in the model group rats were significantly higher than in the control group (*P* < 0.001). The indicators of the AS and XDT groups (0.68, 1.35, and 2.70 g/kg) were significantly lower in the yeast-induced fever model than those of the model group (*P* < 0.05 or *P* < 0.01 or *P* < 0.001, Figure 9). In the yeast-induced fever model, SOD levels were significantly lower than in the control group (*P* < 0.001). The levels of SOD in the AS and XDT groups (0.68 and 2.70 g/kg) were significantly higher in the yeast-induced fever model than those of the model group (*P* < 0.05 or *P* < 0.01, Figure 9). These results suggested that XDT has anti-inflammatory activity by inhibiting the expression of ICAM-1, TNF-*α*, IL-1*β*, MMP-9, and NF-*κ*B; anticoagulant activity by inhibiting the expression of ET-1 and TXB2; and antioxidant activity by increasing the level of SOD and inhibiting the expression of NO to lower blood pressure and improve endothelial function.

#### 3.4.3. Effect of XDT-cs on Glutamate-Induced PC12 Cells

**Figure 10:**
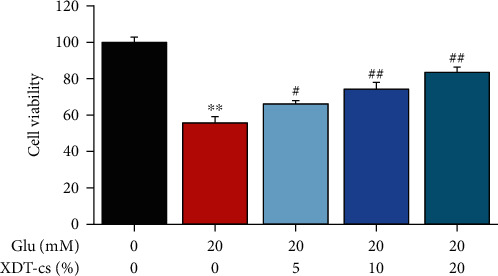
Effect of XDT-cs on the cell viability by CCK-8 assay in response to glutamate-induced PC12 cells. Results were expressed as the mean ± SD (*n* = 3). ^∗∗^*P* < 0.01 vs. the control group; ^#^*P* < 0.05 and^##^*P* < 0.01 vs. the model group.

The effect of XDT-cs on cell viability, in response to glutamate-induced PC12 cells, was measured using CCK-8 assay. The results demonstrated that glutamate treatment reduced the viability of PC12 cells (*P* < 0.01), whereas 5%, 10%, and 20% XDT-cs treatment significantly suppressed the decrease in a dose-dependent manner (*P* < 0.05 or *P* < 0.01, Figure 10). Therefore, it was suggested that XDT-cs can effectively protect PC12 cells from glutamate-induced damage.

**Figure 11:**
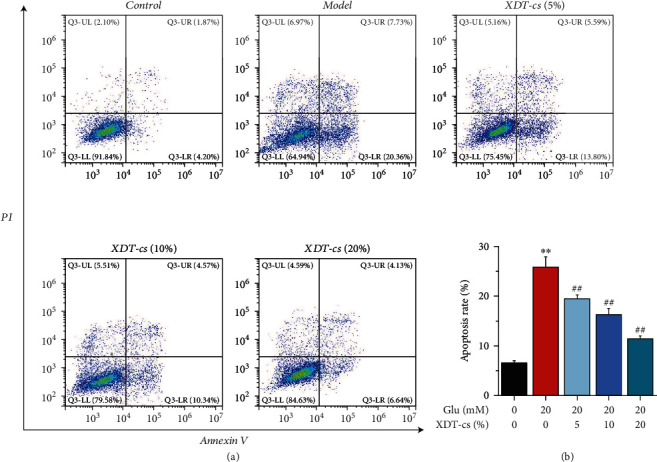
Effect of XDT-cs on the apoptosis by FCM in response to glutamate-induced PC12 cells. The apoptosis rate in PC12 was assessed by FCM analysis after staining with Annexin V and PI. Results were expressed as the mean ± SD (*n* = 3). ^∗∗^*P* < 0.01 vs. the control group; ^#^*P* < 0.05 and^##^*P* < 0.01 vs. the model group.

The effect of XDT-cs on cell apoptosis in glutamate-induced PC12 cells was estimated by FCM analysis after staining with Annexin V and PI. The apoptosis rate was detected by forward light scatter (FSC). The apoptosis rate of PC12 cells was increased by glutamate treatment (*P* < 0.01), whereas 5%, 10%, and 20% XDT-cs treatment significantly suppressed the increase in a dose-dependent manner (*P* < 0.01, Figure 11). These results suggested that XDT-cs can suppress the rate of apoptosis in glutamate-induced PC12 cells.

**Figure 12:**
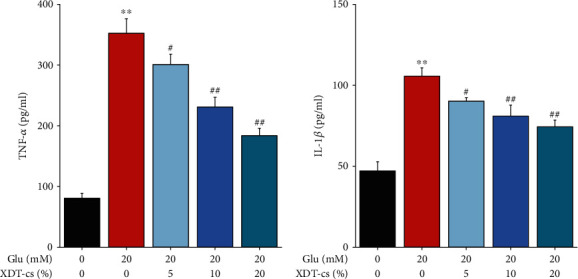
Effect of XDT-cs on the levels of TNF-*α* and IL-1*β* in glutamate-induced PC12 by ELISA. The levels of TNF-*α* (a) and IL-1*β* (b) were normalized to control. Results were expressed as the mean ± SD (*n* = 3). ^∗∗^*P* < 0.01 vs. the control group; ^#^*P* < 0.05 and^##^*P* < 0.01 vs. the model group.

**Figure 13:**
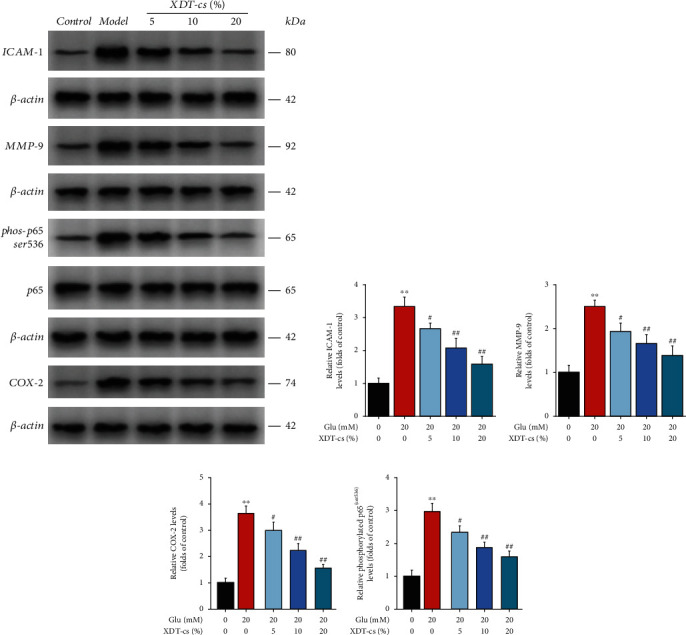
Effect of XDT-cs on the protein level of ICAM-1, MMP-9, COX-2, and p65 in glutamate-induced PC12 by WB assay. Representative bands are shown in (a). The levels of ICAM-1 (b) and MMP-9 (c), and COX-2 (d) and phosphorylation levels of p65 (e) were normalized to the control. Results were expressed as the mean ± SD (*n* = 3). ^∗∗^*P* < 0.01 vs. the control group; ^#^*P* < 0.05 and ^##^*P* < 0.01 vs. the model group.

The anti-inflammatory effect of XDT was further verified using serum pharmacological experiments. Compared to the control group, glutamate treatment increased the levels of TNF-*α*, IL-1*β*, protein levels of ICAM-1, MMP-9, and COX-2, and phosphorylation level of NF-*κ*B (p65) in PC12 cells (*P* < 0.01). However, XDT-cs groups (5%, 10%, and 20%) exhibited a dose-dependent reduction on levels of TNF-*α* and IL-1*β*, protein levels of ICAM-1, MMP-9, and COX-2; and phosphorylation level of NF-*κ*B (p65) in glutamate-induced PC12 cells (*P* < 0.05 or *P* < 0.01, Figures 12 and 13). These results suggested that XDT-cs have anti-inflammatory activity by inhibiting the expression of ICAM-1, TNF-*α*, IL-1*β*, MMP-9, and COX, as well as downregulating the phosphorylation level of NF-*κ*B (p65).

### 3.5. Analysis and Verification of Action Mechanism of XDT

#### 3.5.1. Protein-Protein Interaction Network Analysis

**Figure 14:**
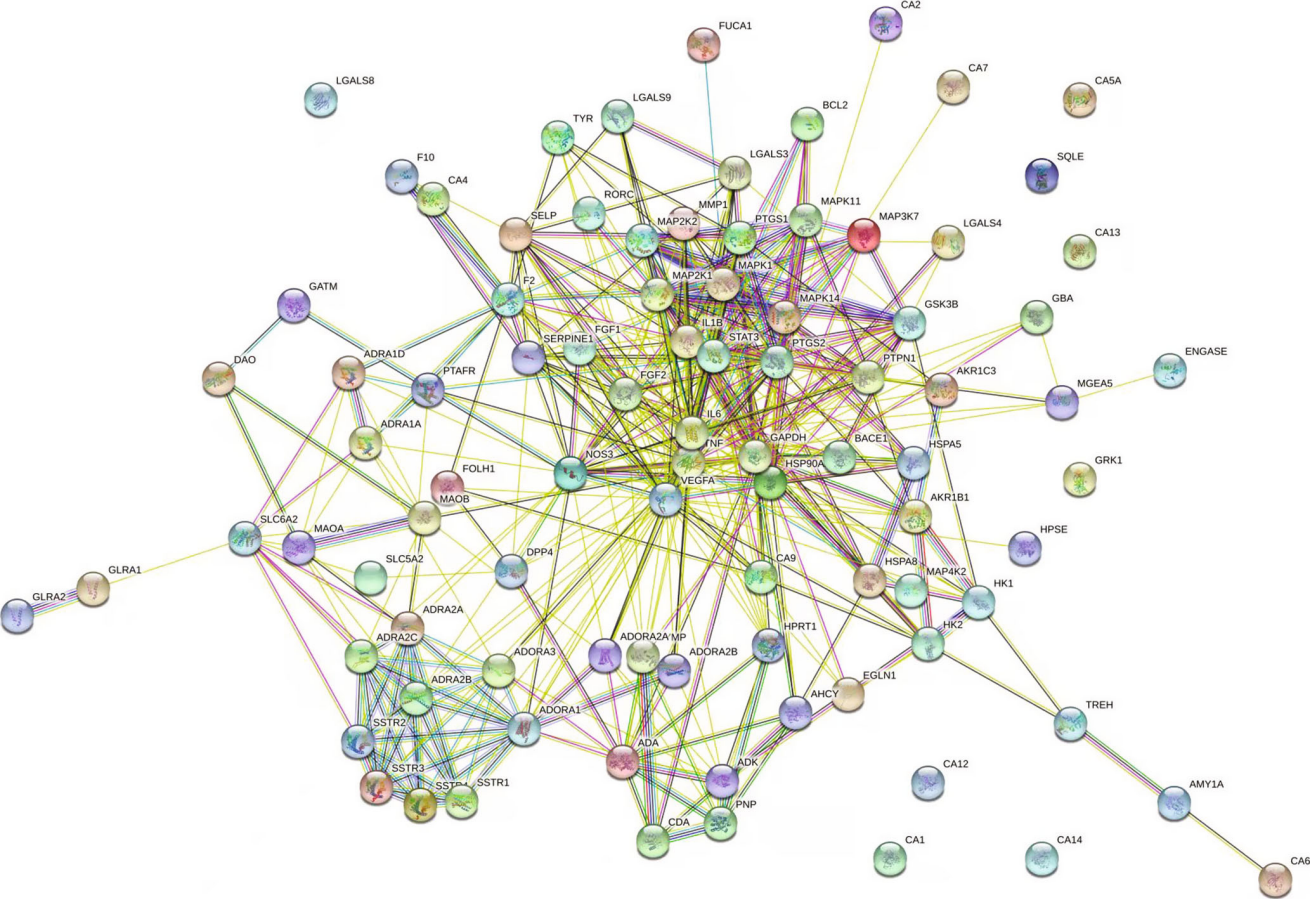
Protein-protein interactions identified in SCXDT by STRING software.

The PPI network was constructed based on the PPIs for 338 targets, which were derived from inner circle 1 and inner circle 2 in the C-T network diagram of SCXDT (Figure 6), to further reveal the action mechanism of the core targets of SCXDT. There were 91 nodes and 448 edges in the PPI network, with an average node degree of 9.85 and a PPI enrichment *P* value < 1.0*e* − 16 (Figure 14). The top 10 targets were TNF, VEGFA, glyceraldehyde-3-phosphate dehydrogenase (GAPDH), IL6, signal transducers, and activators of transcription3 (STAT3), nitric oxide synthase 3 (NOS3), MAPK1, PTGS2, IL1*β*, and heat shock protein 90 alpha family class A member 1 (HSP90AA1). TNF is a multipotent cytokine that participates in the pathogenesis of a series of physiological processes that control inflammation, the antitumor response, and immune system homeostasis, such as proinflammatory reactions including IL-1*β* and IL-6 [15]. MAPK1, which is also known as extracellular regulated protein kinases2 (ERK2), is a highly conserved serine/threonine-protein kinase system that participates in cell proliferation, differentiation, movement, stress response, and other activities, as well as being essential in extracellular signal transduction and cellular response [16]. VEGFA is a rich and powerful angiogenic factor that participates in the regulation of innate immunity [17]. The results suggested that the core targets of SCXDT are critical in inflammation, oxidative stress, coagulation, and apoptosis.

#### 3.5.2. Pathway Enrichment

**Figure 15:**
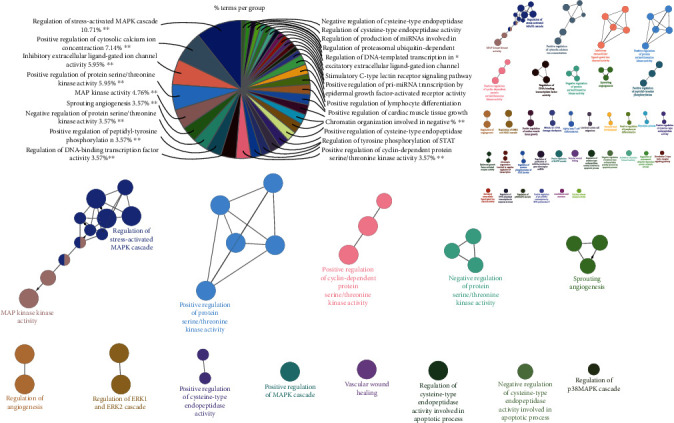
ClueGO functional analysis of all targets related to SCXDT. A functionally grouped network was used to depict the GO terms and pathways representative of target genes; the GO terms are rendered as nodes, and size of the node represents significance. Functionally related groups can partially overlap. Node pie charts represent the reactome analysis of targets. Only the most important term was labeled. The aforementioned GlueGO setting was a representative analysis of the targets.

To explore the biological mechanisms on the targets of SCXDT, Gene Ontology (GO) enrichment analysis of all SCXDT-related targets was performed using ClueGO. The GO terms were classified, and the majority of them involved regulation of stress-activated MAPK cascade (10.71%), positive regulation of cytosolic calciumion concentration (7.14%), inhibitory extracellular ligand-gated ion channel activity (5.95%), positive regulation of protein serine/threonine kinase activity (5.95%), and MAP kinase activity (4.76%) among others (Figure 15). The MAPK signal pathways accounted for the highest proportion, including stress-activated MAPK cascade (10.71%), positive regulation of protein serine/threonine kinase activity (5.95%), negative regulation of protein serine/threonine kinase activity (3.75%), positive regulation of cyclin-dependent protein serine/threonine kinase activity (3.57%), and MAP kinase kinase activity (4.76%). Stress-activated MAPK cascade involves a three-cascade reaction: (Mitogen-activated protein kinase kinase kinase (MAPKKK), Mitogen-activated protein kinase kinase (MAPKK), and Mitogen-activated protein kinase (MAPK). Positive regulation of protein serine/threonine kinase activity, negative regulation of protein serine/threonine kinase activity, and positive regulation of cyclin-dependent protein serine/threonine kinase activity belong to the serine/threonine-protein kinase (serine/threonine kinase), which is also known as MAPK. The MAPK signaling pathway regulates inflammatory, neuroprotection, and oxidative stress, among others. There are also certain pathways, including spring angiogenesis, regulation of cysteine-type endopeptidase activity involved in apoptotic process, and vascular wound healing. The signal pathways regulated by SCXDT targets are involved in inflammation, oxidative stress, coagulation, cell proliferation and differentiation, apoptosis, energy metabolism, and other processes, revealing the complexity of the SCXDT pathological process in BHSS-related disease.

**Figure 16:**
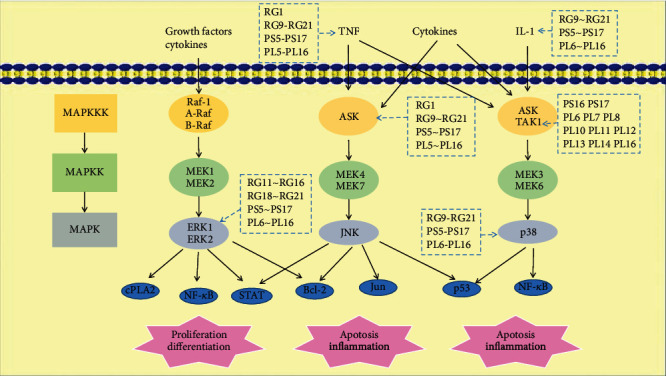
SCXDT and hitting targets on MAPK signal pathways. BB: *Bubalus bubalis* Linnaeus; RG: *Rehmannia glutinosa* Libosch.; PL: *Paeonia lactiflora* Pall.; PS: *Paeonia suffruticosa* Andr.

There are three main classical MAPKs with different isoforms ERKs (with ERK1 and ERK2 isoforms), JNKs (c-Jun N-terminal kinases, with JNK1, JNK2, and JNK3 isoforms), and p38 MAPKs (with p38*α*, p38*β*, p38*γ*, and p38*δ* isoforms).  Figure 16 shows that there are more SCXDT on the p38MAPK signal pathways, based on the predicted results of the SCXDT and targets (Table S2).

#### 3.5.3. Effect of SCXDT on the p38MAPK Signaling Pathway

**Figure 17:**
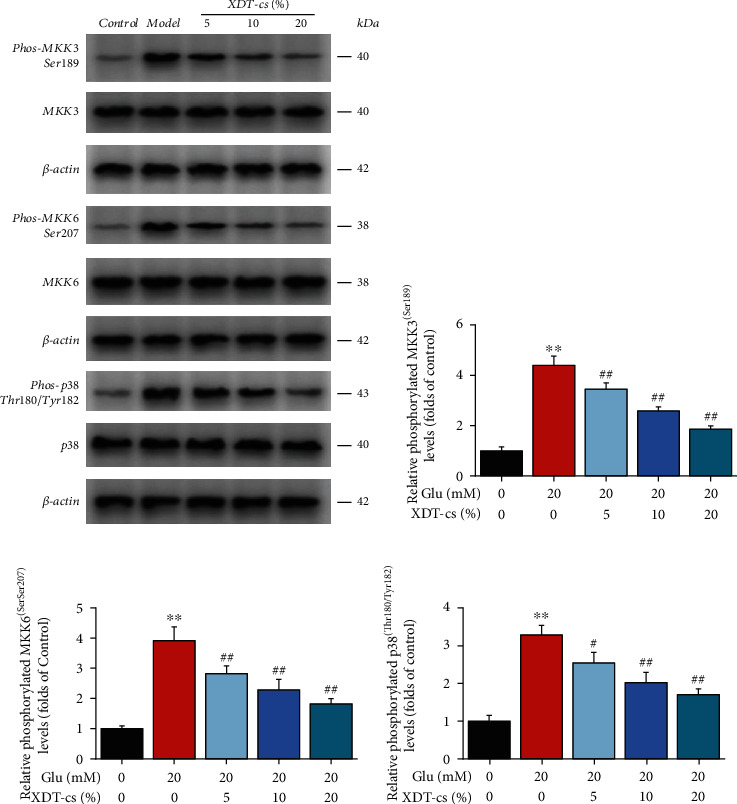
Representative western blots showing effect of XDT-cs on expression levels of MKK3, MKK6, and p38 proteins in glutamate-treated PC12 cells (a). Phosphorylation levels of MKK3 (b), MKK6 (c), and p38 (d). Data are presented as the mean ± SD (*n* = 3). ^∗∗^*P* < 0.01 vs. the control group; ^#^*P* < 0.05 and^##^*P* < 0.01 vs. the model group.

To verify the effect of XDT-cs on the p38MAPK signaling pathway, the protein expression levels of MKK3, MKK6, and p38 via the p38MAPK signaling pathway were measured in glutamate-injured PC12 cells (Figure 17). Compared to the control group, the phosphorylation level of MKK3, MKK6, p38 of the model group increased significantly (*P* < 0.01), but decreased significantly in a dose-dependent manner (*P* < 0.05 or *P* < 0.01) in the XDT-cs groups (5%, 10%, and 20%). It was proposed that XDT-cs significantly downregulate protein expression of MKK3, MKK6, and p38 in the p38MAPK signaling pathway.

## 4. Discussion

A TCM syndrome was a clear characteristic of all clinical manifestations in one patient. According to TCM, BHSS is caused by an inflammatory response (either infectious or noninfectious) in the tissues and blood stagnation. Clinically, BHSS patients exhibit elevated body temperature and abnormal blood rheological indexes, which primarily manifest as abnormal inflammation and cytokine expression, abnormal vascular endothelial cell function, platelet function, and blood rheological indexes, as well as imbalance of coagulation and fibrinolysis [3]. Yeast-induced fever rat models present an excellent platform for understanding entry of exogenous pyrogens into the body, stimulation of immune cells and endogenous pyrogens, such as IL-1*β*, TNF-*α*, IL-6, and other thermogenic cytokines that regulate the temperature regulation centers through neuronal mediators, causing fever, blood rheology, and abnormalities of coagulation factors [4, 18]. Results of the present study revealed that yeast-induced fever rats had pathology of and characteristic profile on body temperature and abnormal blood rheological indexes that were consistent with the clinical manifestations observed in one BHSS patient. Therefore, we adopted the yeast-induced fever rat model with BHSS to evaluate the effect of XDT. Results showed that XDT caused significantly better antipyretic and anticoagulant effects on BHSS-related disease compared to AS.

Numerous BHSS-related clinical diseases, such as acute cerebral hemorrhage, toad epilepsy, Alzheimer's disease, malignant glioma, Parkinson's disease, and migraine, are closely related to nerve damage involving excitatory amino acid toxicity. Previous studies have shown that high levels of glutamate, which is the main excitatory neurotransmitter of the central nervous system, induce hyperexcitable glutamate receptors, thereby causing glutamate excitotoxicity which subsequently triggers a cascade of events that eventually lead to apoptosis or necrosis and elevated levels of inflammatory mediators [19]. Therefore, glutamate-induced PC12 cells represent an ideal cell-based system for investigating the effects and underlying mechanisms of XDT on neuroprotection for treatment of BHSS-related diseases. Notably, XDT-cs promotes cell viability, reduces cellular apoptosis and downregulates levels of TNF-*α*, IL-1*β*, ICAM-1, and MMP9 in glutamate-induced PC12, suggesting that XDT exerts a neuroprotective effect on glutamate-induced PC12 cells by inhibiting inflammation.

In the present study, we first used HPLC-QTOF/MS/MS to identify 60 SCXDT in yeast-induced fever rats, then constructed a C-T network based on SCXDT and predicted targets. We predicted a total of 19 core constituents in SCXDT, based on a screening degree value greater than or equal to 17, and experimentally verified the neuroprotective effects of 9 of them using glutamate-induced PC12 cells. Among them, paeoniflorin markedly inhibited inflammation and suppressed production of inflammatory medium, thereby protecting PC12 cells from glutamate-induced damage by inhibiting apoptosis, alleviating thrombosis by up-regulation of urokinase-type plasminogen activator via the MAPK signaling pathway [20, 21]. Adenosine has been shown to regulate inflammatory cells and vascular endothelial growth factors via its receptors, to abrogate glutamate-induced cytotoxicity in PC12 through A1AR positive allosteric modulation [22, 23]. On the other hand, geniposide protects cells against hypoxia/reperfusion-induced blood-brain barrier impairment by mitigating the release of inflammatory cytokines and increasing release of brain-derived neurotrophic as well as glial cell-derived neurotrophic factors. Moreover, it reportedly downregulates expression of HIF-1*α*-independent VEGF and angiogenesis thereby inhibiting the TLR4/MyD88 signaling pathway [24, 25]. Aucubin has been shown to alleviate inflammation and H_2_O_2_-induced neuron cell apoptosis through Nrf2-mediated signaling activity, to exert proangiogenic effects through the ER*β*-mediated VEGF signaling pathway [26, 27]. On the other hand, salidroside was found to attenuate neuroinflammation and alleviate apoptosis in PC12 cells, thereby suppressing complications of brain ischemic injury [28]. Guanosine not only exerts neuroprotective effects and reduces inflammatory response but also exhibits antiplatelet and antithrombotic properties through adenosine-related cAMP-PKA signaling [29, 30], while paeonol reportedly attenuates inflammatory and coagulation reactions thereby alleviating PC12 apoptosis by modulating downregulation of ERK activation [31, 32]. Catapol was found to exert neuroprotective effects against acute focal ischemic stroke by inhibiting apoptosis and suppressing inflammatory reactions, thereby protecting vascular structure, and promoting angiogenesis in focal cerebral ischemic rats. Notably, it exerts these effects by regulating the HIF-1*α*/VEGF pathway [33, 34]. Previous studies have shown that oxypaeoniflorin attenuates inflammation with lower levels of inflammatory cytokines, exerts anticoagulant activity by upregulating HSP-70 and coronin-1B expression, and reduces the ratio of adhesion platelets, thereby generating neuroprotective effects by modulating the cAMP/PKA/CREB signaling pathway [35, 36]. Among the 20 SCXDT, rehmapicroside exerts neuroprotective effects to inhibit mitophagy, attenuates apoptotic cell death, reduces infarct sizes, and improves neurological functions during cerebral ischemia-reperfusion injury [37]. Taken together, these findings indicate that apart from anticoagulant activity, the core constituents predicted by network pharmacology in SCXDT exert both neuroprotective and anti-inflammatory effects.

Further analysis of the C-T network revealed that 60 SCXDT acted on 338 targets, indicating that XDT has excellent properties that make it a multicomponent and multitarget against BHSS-related disease. Furthermore, the SCXDT targets predicted by the C-T-D network were mainly involve in biological processes related to inflammation, oxidative stress, coagulation, and cell apoptosis, among others. Next, we evaluated the effect of XDT and XDT-cs extracts on yeast-induced fever rats with BHSS and glutamate-induced PC12 cells and found that XDT not only significantly downregulated expression of TNF-*α*, IL-1, NF-*κ*b, COX-2, ICAM-1, MMP-9, ET-1, NO, SOD, and TXB2 but also downregulated phosphorylation of p65 and had a neuroprotective effect. Previous studies have shown that during inflammation, chronic stress antigens, such as NF-*κ*B, IL-6, TNF-*α*, IL-1, and IL-10, stimulate microglia (resident immune cells) and astrocytes to activate the typical inflammatory pathways thereby causing neuronal injury [38]. Notably, MMP-9 has been closely associated with blood stasis, by creating several interactions with the coagulation cascade, while ET-1 and TXB2 reportedly regulate cardiovascular and platelet function to induce stasis [39, 40]. Additional research evidences have shown that activation of NO, including neuronal NOS, iNOS, and eNOS, is accompanied by development of oxidative stress, which causes unflipping of NOS to exacerbate oxidative/nitrogenated stress. SOD, an enzymatic scavenger and the first line of defense against ROS accumulation, plays a crucial role in limiting oxidative damage, and its activity is associated with antioxidant response [41]. On the other hand, transcription factor NF-*κ*B is a central mediator of inflammation with multiple links to thrombotic processes [42]. Our experimental results confirmed that XDT exerts inhibitory effects on inflammation, oxidative stress, coagulation, cell apoptosis, and nerve injury, suggesting its potential for the treatment of BHSS-related diseases.

Previous studies have demonstrated that activation of the p38MAPK signaling pathway is not only clinically closely related to development of BHSS-related diseases, but it also plays a crucial role in the inflammatory response, platelet activation, and thrombosis [43]. Notably, inflammatory cytokines, such as TNF-*α* and IL-1*β*, which are upregulated following intracerebral and subarachnoid hemorrhage, activate the p38MAPK signaling pathway in the arterial wall, leading to vasospasm, brain edema, neurobehavioral damage, and inflammation enhancement and inducing apoptosis [44, 45]. More evidence has shown that the p38MAPK signaling pathway also plays a significant role in hemostasis and thrombosis [46]. Results from our PPI network and GO enrichment analysis of all targets related to SCXDT revealed that the p38MAPK signaling pathway occupied the largest proportion. Moreover, our results indicated that XDT significantly downregulated phosphorylation of MKK3, MKK6, and p38 proteins in the p38MAPK signaling pathway. Furthermore, it significantly downregulated expression of TNF-*α*, ICAM-1, IL-1*β*, NF-*κ*B, and MMP-9 activated by the p38MAPK signaling pathway. Collectively, these results suggested that XDT exerts neuroprotective, antioxidant, anticoagulant, and anti-inflammation effects by regulating the p38MAPK signaling pathway for the treatment of BHSS-related disease.

In the present study, we adopted serum medicinal chemistry, in combination with network pharmacology, to identify active components and therapeutic targets of XDT that have potential for treatment of BHSS-related diseases. However, the underlying mechanism of BHSS action is complex involving inflammation, coagulation, cell proliferation and apoptosis, oxidative contingencies, and neuroprotection and dozens of signaling pathways. Further investigations are also needed to comprehensively elucidate the complex mechanisms underlying development and progression of BHSSS-related diseases as well as the signaling pathways involved in the processes.

## 5. Conclusions

In the present study, we used serum pharmacochemistry in combination with network pharmacology to provide the first report on the effect of XDT's active constituents and the underlying mechanism of action in treatment of BHSS-related diseases. A total of 20 important active constituents were identified in XDT and screened. Summarily, XDT played an integrated role in BHSS-related disease by modulating expression of a series of targets, such as IL-1*β*, IL-6, TNF, NOS3, MAPK1, STAT3, and VEGFA, as well as dozens of signaling pathways, key among them the p38MAPK signaling pathway. Furthermore, it exerted significant effects on various biological processes, such as inflammation, coagulation, cell proliferation and apoptosis, oxidative contingencies, and neuroprotection. Taken together, these findings provide invaluable insights to guide future development of effective TCM-based therapies for treatment of BHSS-related diseases.

## Figures and Tables

**Table 1:** The core constituent, degree value, and structure of SCXDT.

Number	ID	PubChem CID	Molecule name	Degree	Structure
1	BB5	60961	Adenosine	75	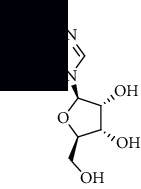
2	RG6	107848	Geniposide	56	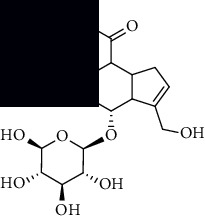
3	PL3	21631108	Moudanpioside F	52	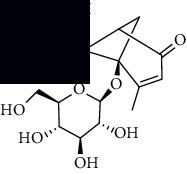
4	BB1	135398635	Guanosine	48	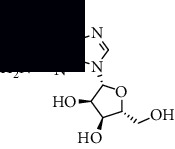
5	PS3	21631103	Mudanpioside D	45	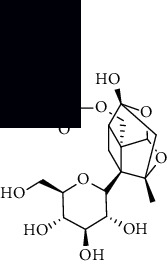
6	PS4	19844	Methyl vanillate	42	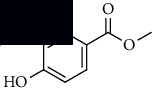
7	PL2	8814958	1-O-*β*-D-Glucopyranosyl-paeonisuffron	40	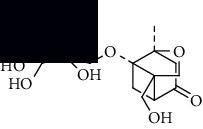
8	PL1	71452333	8-Debenzoylpaeoniflorin	32	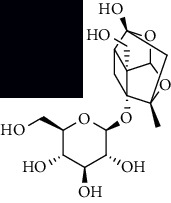
9	BB2	5960	Aspartic acid	30	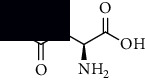
10	RG7	21637711	Rehmapicroside	28	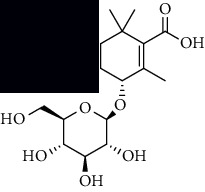
11	PL5	442534	Paeoniflorin	28	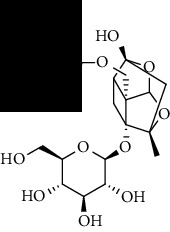
12	PS1	10592506	Mudanoside A	28	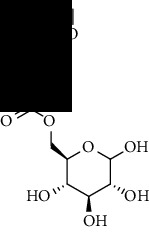
13	PS2	11092	Paeonol	28	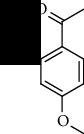
14	BB3	145742	Proline	26	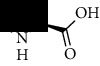
15	RG1	91458	Aucubin	26	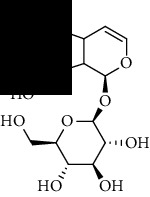
16	RG5	159278	Salidroside	23	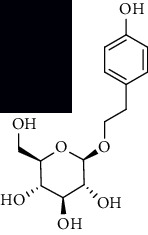
17	PL4	21631105	Oxypaeoniflora	23	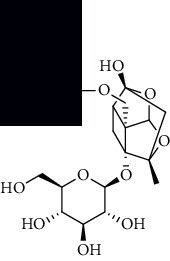
18	RG3	91520	Catalpol	20	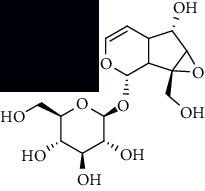
19	RG2	158144	8-Epiloganic acid	17	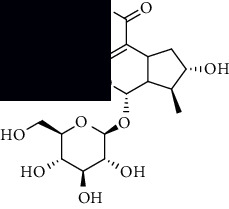

Core constituents in SCXDT with values greater than or equal to 17 are listed in the above table according to the degree of the constituents and targets, BB, *Bubalus bubalis* Linnaeus, RG, *Rehmannia glutinosa* Libosch., PL, *Paeonia lactiflora* Pall., PS, *Paeonia suffruticosa* Andr., PubChem CID, the compound identifier of PubChem, degree, connection value of constituents, and targets.

## Data Availability

The data used to support the findings of this study are included within the article and the supplementary information files.

## Supplementary Materials

Supplementary Materials Table S1: HPLC-QTOF/MS/MS analysis of SCXDT. Table S2: compound targets for each serum compound of XDT.
