# Supplementary material for: Serum Pharmacochemistry Combining Network Pharmacology to Discover the Active Constituents and Effect of Xijiao Dihuang Tang Prescription for Treatment of Blood-Heat and Blood-Stasis Syndrome-Related Disease
Source: Oxid Med Cell Longev. 2022 Feb 7;2022:6934812. doi: 10.1155/2022/6934812 (PMC8845118; doi:10.1155/2022/6934812)
Supplement: Supplementary Materials — Table S1: HPLC-QTOF/MS/MS analysis of SCXDT. Table S2: compound targets for each serum compound of XDT. [file 6934812.f1.zip › table s1.docx]

**Supplementary Table 1A (Tab. S1A) Identification results of constituents of extract and serum samples of XDT in negative mode by HPLC-Q-TOF-MS.**

| No | T_R_  (min) | ESI^-^ （m/z） | | MW | MF | Name | Detected | | Source | Reference |
| --- | --- | --- | --- | --- | --- | --- | --- | --- | --- | --- |
|  |  | MS | MS/MS |  |  |  | Vitro | Serum |  |  |
| 1 | 11.33 | 749 | 387.01,305.02,223.01,141.02,89.03 | 750 | C_27_H_42_O_22_S | Rehmannioside D sulfate | - | + | RG | [[1](#_ENREF_1)] |
| 2 | 11.38 | 695 | 449.02,367.04,285.03,203.02,171.03 | 696 | C_30_H_32_O_17_S | Galloyl paeoniflorin sulfate | - | + | PL | [[2](#_ENREF_2)] |
| 5 | 12.60 | 363 | 201.08,171.07,125.06,71.02,59.02 | 364 | C_15_H_24_O_10_ | Dihydrocatalpol | + | - | RG | [[1](#_ENREF_1)] |
| 5a | 12.62 | 381 | 331.00,217.00,151.00 | 382 | C_15_H_10_O_10_S | Quercetin 3'-sulfate | - | + | PS | [[3](#_ENREF_3)] |
| 5b | 12.63 | 807 | 545.02,463.01,299.01,212.06,141.00 | 808 | C_36_H_40_O_21_ | Galloyl paeoniflorin glucuronide | - | + | PL | [[2](#_ENREF_2)] |
| 5c | 12.86 | 277 | 197.04,182.02,181.45,123.01 | 278 | C_9_H_10_O_8_S | Syringic acid sulfate | - | + | PS | [[4](#_ENREF_4)] |
| 5d | 12.87 | 543 | 311.12,301.06,297.10,215.09,141.01 | 544 | C_18_H_24_O_17_S | Mudanoside B sulfate | - | + | PS | [[5](#_ENREF_5)] |
| 6 | 12.88 | 375 | 345.12,195.06,177.06,165.05,89.02 | 376 | C_16_H_24_O_10_ | 8-Debenzoylpaeoniflorin | + | + | PL | [[1](#_ENREF_1)] |
| 6a | 12.91 | 663 | 581.04,499.05,417.03,335.03,171.03 | 664 | C_30_H_32_O_15_S | Benzoyloxypaeoniflorin sulfate | - | + | PL | [[2](#_ENREF_2)] |
| 8 | 12.95 | 345 | 183.07,179.05,165.05 | 346 | C_15_H_22_O_9_ | Aucubin | + | + | RG | [[1](#_ENREF_1)] |
| 9 | 12.98 | 375 | 345.12,213.08,195.07,177.06,165.06 | 376 | C_16_H_24_O_10_ | 8-Epiloganic acid | + | + | RG | [[1](#_ENREF_1)] |
| 10 | 13.56 | 493 | 331.07,179.06,169.01,89.03 | 494 | C_19_H_26_O_15_ | Galloylsucrose | + | - | PL | [[5](#_ENREF_5)] |
| 10a | 13.62 | 851 | 611.10,393.06,223.02,212.06,141.02 | 852 | C_34_H_28_O_24_S | 1,2,3,6-Tetragalloyl glucose sulfate | - | + | PL | [[2](#_ENREF_2)] |
| 10b | 13.95 | 289 | 229.08,207.09,101.03 | 290 | C_11_H_14_O_7_S | Genipin sulfate | - | + | PS | [[6](#_ENREF_6)] |
| 11 | 14.37 | 685 | 263.08,181.05,179.06, 89.02 | 686 | C_27_H_42_O_20_ | Rehmannioside D | + | - | RG | [[1](#_ENREF_1)] |
| 12 | 14.79 | 523 | 181.05,179.06,119.04,89.03,59.02 | 524 | C_21_H_32_O_15_ | Melittoside | + | - | RG | [[1](#_ENREF_1)] |
| 13* | 14.85 | 282 | 150.04,133.02,108.02,66.01,107.04 | 283 | C_10_H_13_N_5_O_5_ | Guanosine | + | + | BB | - |
| 14 | 14.96 | 359 | 197.08,179.07,122.04, 85.03 | 360 | C_16_H_24_O_9_ | 1-O-β-D-glucopyransoyl-paeonisuffron | + | + | PL | [[7](#_ENREF_7)] |
| 15 | 15.09 | 463 | 403.09,343.07,241.04,169.01 | 464 | C_18_H_24_O_14_ | Mudanoside B | + | - | PS | [[5](#_ENREF_5)] |
| 16* | 15.14 | 361 | 209.07,199.06,169.01,165.00 | 362 | C_15_H_22_O_10_ | Catalpol | + | + | RG | - |
| 16a | 15.22 | 701 | 505.11,423.10,223.01,212.07 | 702 | C_30_H_38_O_19_ | Mudanpioside E glucuronide | - | + | PL | [[2](#_ENREF_2)] |
| 16b | 15.96 | 441 | 359.17,179.01, 88.04, | 442 | C_16_H_26_O_12_S | Lamiol sulfate | - | + | RG | [[8](#_ENREF_8)] |
| 17 | 16.22 | 329 | 167.03,108.02 | 330 | C_14_H_18_O_9_ | Mudanoside A | + | + | PS | [[2](#_ENREF_2)] |
| 17a | 16.47 | 863 | 803.19,803.06,623.06,487.00,387.03 |  | C_36_H_48_O_22_S | Jionoside A1 sulfate | - | + | RG | [[1](#_ENREF_1)] |
| 17b | 16.54 | 495 | 465.14,232.97,165.05,137.02 | 496 | C_23_H_28_O_12_ | Oxidation albiflorin | - | + | PL | [[9](#_ENREF_9)] |
| 18* | 16.57 | 373 | 211.06,149.06,123.05,105.03,59.02 | 374 | C_16_H_22_O_10_ | Geniposidic acid | + | + | RG | - |
| 18a | 16.61 | 495 | 465.14,333.10,177.06,137.02 | 496 | C_23_H_28_O_12_ | Oxymudanpioside I | - | + | PL | [[10](#_ENREF_10)] |
| 18b | 16.62 | 373 | 197.05,182.02,167.00,123.01,59.02 | 374 | C_15_H_18_O_11_ | Syringic acid glucuronide | - | + | PS | [[4](#_ENREF_4)] |
| 19 | 16.80 | 461 | 315.11,161.05,135.04 | 462 | C_20_H_30_O_12_ | Decaffeoyl verbascoside | + | - | RG | [[1](#_ENREF_1)] |
| 20 | 17.04 | 487 | 179.03,135.05,133.03 | 488 | C_21_H_28_O_13_ | Cistanoside F | + | - | RG | [[8](#_ENREF_8)] |
| 21 | 17.36 | 635 | 483.08,465.07,313.06,169.01,125.02 | 636 | C_27_H_24_O_18_ | Trigalloyl glucose | + | - | PS | [[2](#_ENREF_2)] |
| 22* | 17.50 | 289 | 245.08,203.07,151.04,109.03 | 290 | C_15_H_14_O_6_ | Catechin | + | - | PL | - |
| 23 | 17.54 | 343 | 181.09,163.08,151.08,136.05,109.07 | 344 | C_16_H_24_O_8_ | Mudanpioside F | + | + | PL | [[5](#_ENREF_5)] |
| 24* | 17.67 | 299 | 137.02,136.02,89.02,59.02 | 300 | C_14_H_20_O_7_ | Salidroside | + | + | RG | - |
| 24a | 17.73 | 201 | 139.07,99.01,89.02,71.05,61.99 | 202 | C_9_H_14_O_5_ | Deglycosylation product of dihydrocatalpol | - | + | RG | [[1](#_ENREF_1)] |
| 25* | 17.82 | 495 | 333.10,177.06,165.06,137.02 | 496 | C_23_H_28_O_12_ | Oxypaeoniflorin | + | + | PL | - |
| 26 | 17.85 | 785 | 623.23,161.02 | 786 | C_35_H_46_O_20_ | Echinacoside | + | - | RG | [[1](#_ENREF_1)] |
| 27 | 18.02 | 525 | 495.15,181.05,167.03,123.05 | 526 | C_24_H_30_O_13_ | Mudanpioside E | + | - | PL | [[2](#_ENREF_2)] |
| 28 | 18.03 | 787 | 635.09,617.08,465.07,295.05,169.01 | 788 | C_34_H_28_O_22_ | 1,2,3,6-Tetragalloyl glucose | + | - | PL | [[2](#_ENREF_2)] |
| 29 | 18.14 | 611 | 445.10,301.06,283.04, 169.01 | 612 | C_27_H_32_O_16_ | Suffruticoside B | + | - | PS | [[5](#_ENREF_5)] |
| 29a | 18.25 | 483 | 319.07,241.04,202.00,180.07,148.04 | 484 | C_20_H_20_O_14_ | 1,6-Bis-O-galloyl-beta-D-glucose | - | + | PS | [[2](#_ENREF_2)] |
| 30* | 18.29 | 939 | 769.10,617.08,601.09, 47.06,169.01 | 940 | C_41_H_32_O_26_ | 1,2,3,4,6-Pentagalloyl glucose | + | - | PL | - |
| 31 | 18.46 | 799 | 623.22,605.21,193.05,175.04,160.02 | 800 | C_36_H_48_O_20_ | Jionoside A1 | + | - | RG | [[1](#_ENREF_1)] |
| 32 | 18.51 | 799 | 623.22,605.21,193.05,175.04,160.02 | 800 | C_36_H_48_O_20_ | Cistanoside A | + | - | RG | [[11](#_ENREF_11)] |
| 33 | 18.72 | 377 | 197.08,179.06,153.09, 89.03, 59.02 | 378 | C_16_H_26_O_10_ | Lamiol | + | - | RG | [[8](#_ENREF_8)] |
| 34 | 18.86 | 475 | 223.06,161.05,113.02,89.02 | 476 | C_21_H_32_O_12_ | Darendoside B | + | - | RG | [[8](#_ENREF_8)] |
| 35 | 18.89 | 611 | 445.10,169.01 | 612 | C_27_H_32_O_16_ | Suffruticoside A | + | - | PS | [[5](#_ENREF_5)] |
| 36 | 19.05 | 611 | 445.10,169.01,165.05,150.03 | 612 | C_27_H_32_O_16_ | Suffruticoside D | + | - | PS | [[5](#_ENREF_5)] |
| 36a | 19.06 | 215 | 197.09,197.07,153.09 | 216 | C_10_H_16_O_5_ | Deglucose product of lamiol | - | + | RG | [[8](#_ENREF_8)] |
| 37 | 19.15 | 611 | 445.10,169.01,165.06,150.03 | 612 | C_27_H_32_O_16_ | Suffruticoside C | + | - | PS | [[8](#_ENREF_8)] |
| 38 | 19.45 | 623 | 461.17,315.11,179.03,161.02 | 624 | C_29_H_36_O_15_ | Acteoside | + | - | RG | [[1](#_ENREF_1)] |
| 39 | 19.49 | 315 | 813.29,637.24,193.05,175.04,160.02 | 316 | C_37_H_50_O_20_ | Jionoside B1 | + | - | RG | [[1](#_ENREF_1)] |
| 40 | 19.60 | 813 | 641.21,611.20,489.16,293.13,121.03 | 814 | C_29_H_38_O_16_ | 6'-O-β-D-glucopyranosylalbiflorin | + | - | PL | [[12](#_ENREF_12)] |
| 41* | 19.74 | 387 | - | 388 | C_17_H_24_O_10_ | Geniposide | + | + | PL | - |
| 42* | 19.95 | 225 | - | 226 | C_11_H_14_O_5_ | Genipin | + | - | PS | - |
| 43* | 19.97 | 479 | 313.06,169.01,165.05,121.03 | 480 | C_23_H_28_O_11_ | Albiflorin | + | - | PL | - |
| 44 | 19.98 | 345 | 179.06,165.09,89.03,59.02 | 346 | C_16_H_26_O_8_ | Rehmapicroside | + | + | RG | [[1](#_ENREF_1)] |
| 45* | 20.02 | 479 | 327.11,169.01,165.05,121.03 | 480 | C_23_H_28_O_11_ | Paeoniflorin | + | + | PL | - |
| 46 | 20.10 | 623 | 461.17,315.11,179.03,161.02 | 624 | C_29_H_36_O_15_ | Isoacteoside | + | - | RG | [[1](#_ENREF_1)] |
| 47 | 20.21 | 631 | 613.16,399.09,313.06,169.01 | 632 | C_30_H_32_O_15_ | Galloyl paeoniflorin | + | - | PL | [[2](#_ENREF_2)] |
| 48 | 20.50 | 615 | 431.14,281.07,239.06,137.02 | 616 | C_30_H_32_O_14_ | Mudanpioside H | + | - | PS | [[2](#_ENREF_2)] |
| 49 | 20.54 | 197 | 169.01,125.02,124.02 | 198 | C_9_H_10_O_5_ | Ethyl gallate | + | - | PL | [[13](#_ENREF_13)] |
| 50 | 20.56 | 197 | 169.01,125.02,124.02,78.01 | 198 | C_9_H_10_O_5_ | Syringic acid | + | - | PS | [[14](#_ENREF_14)] |
| 51 | 20.59 | 335 | 168.01,140.01,124.02 | 336 | C_15_H_12_O_9_ | Galloyl methyl gallate | + | - | PS | [[15](#_ENREF_15)] |
| 52 | 20.95 | 167 | 108.02 | 168 | C_8_H_8_O_4_ | Vanillic acid | + | + | RG | [[8](#_ENREF_8)] |
| 53 | 21.16 | 651 | 475.18,193.05,175.04,160.02 | 652 | C_31_H_40_O_15_ | Martynoside | + | - | RG | [[1](#_ENREF_1)] |
| 54 | 21.39 | 181 | 629.19,461.15,459.13,311.08,167.03 | 182 | C_9_H_10_O_4_ | Methyl vanillate | + | + | PS | [[15](#_ENREF_15)] |
| 55 | 21.50 | 479 | 357.12,177.05,121.03 | 480 | C_23_H_28_O_11_ | Mudanpioside I | + | - | PL | [[10](#_ENREF_10)] |
| 57 | 21.53 | 599 | 477.14,281.07,137.02,121.03 | 600 | C_30_H_32_O_13_ | Benzoyloxypaeoniflorin | + | - | PL | [[2](#_ENREF_2)] |
| 58 | 21.70 | 629 | 461.15,459.13,311.08,167.03 | 630 | C_31_H_34_O_14_ | Mudanpioside J | + | - | PS | [[2](#_ENREF_2)] |
| 59 | 21.74 | 599 | 447.13,431.13,281.07,137.02,121.03 | 600 | C_30_H_32_O_13_ | Mudanpioside C | + | - | PS | [[2](#_ENREF_2)] |
| 60 | 21.94 | 523 | 463.22,193.05,134.04,121.03 | 524 | C_25_H_32_O_12_ | 6-O-E-Feruloylajugol | + | - | RG | [[1](#_ENREF_1)] |
| 61 | 23.05 | 301 | 179.00,151.00,121.02,65.00 | 302 | C_15_H_10_O_7_ | Quercetin | + | - | PS | [[3](#_ENREF_3)] |
| 62 | 23.07 | 121 | 93.03,77.04 | 122 | C_7_H_6_O_2_ | Benzoic acid | + | - | PL | [[16](#_ENREF_16)] |
| 63 | 23.15 | 583 | 431.13,165.06,121.03 | 584 | C_30_H_32_O_12_ | Benzoylpaeoniflorin | + | - | PL | [[2](#_ENREF_2)] |
| 64 | 23.42 | 271 | 151.00,119.05,93.03,65.00 | 272 | C_15_H_12_O_5_ | Naringenin | + | - | RG | [[17](#_ENREF_17)] |
| 65* | 23.52 | 165 | 150.03,122.04,108.02 | 166 | C_9_H_10_O_3_ | Paeonol | + | + | PS | - |
| 65a | 23.60 | 463 | 283.26,267.13,185.13 | 464 | C_18_H_24_O_14_ | Deglycosylation product of suffruticoside A | - | + | PS | [[5](#_ENREF_5)] |
| 65b | 23.81 | 347 | 265.10,203.08,137.02,121.03 | 348 | C_15_H_24_O_9_ | Deglucuronide product of 6-O-E- Feruloylajugol | - | + | RG | [[1](#_ENREF_1)] |
| 65c | 23.86 | 375 | 357.20,243.15,141.02,79.96 | 376 | C_16_H_24_O_10_ | Deglycosylation product of mudanpioside I | - | + | PL | [[10](#_ENREF_10)] |
| 65d | 25.04 | 463 | 283.26,283.11,159.09 | 464 | C_18_H_24_O_14_ | Deglycosylation product of suffruticoside D | - | + | PS | [[5](#_ENREF_5)] |
| 66 | 25.07 | 509 | 481.23,449.23,327.21,159.02 | 510 | C_24_H_30_O_12_ | Mudanpioside D | + | + | PS | [[15](#_ENREF_15)] |
| 66a | 25.98 | 463 | 445.22,365.13,245.09,130.07 | 464 | C_18_H_24_O_14_ | Deglycosylation product of suffruticoside B | - | + | PS | [[5](#_ENREF_5)] |
| 66b | 26.42 | 239 | 177.10,149.01,123.08,83.06,71.01 | 240 | C_12_H_16_O_5_ | Methylated genipin | - | + | PS | [[6](#_ENREF_6)] |
| 66c | 27.19 | 341 | 281.26,177.05,133.07 | 342 | C_15_H_18_O_9_ | Deglycosylation product of cistanoside F | - | + | RG | [[8](#_ENREF_8)] |
| 66d | 28.49 | 539 | 397.21,315.19,313.18,227.10,89.03 | 540 | C_25_H_32_O_13_ | Methylated mudanpioside E | - | + | PL | [[2](#_ENREF_2)] |
| 66e | 29.55 | 463 | 445.23,283.26,283.11,201.11,125.10 | 464 | C_18_H_24_O_14_ | Deglycosylation product of suffruticoside C | - | + | PS | [[5](#_ENREF_5)] |
| 66f | 33.66 | 787 | 786.86,676.71,676.20,617.21 | 788 | C_34_H_28_O_22_ | Deglycosylation product of 1,2,3,4,6-pentagalloyl glucose | - | + | PL | [[2](#_ENREF_2)] |
| 67a | 39.08 | 501 | 618.25,325.15,164.06 | 502 | C_22_H_30_O_13_ | Methylated cistanoside F | - | + | RG | [[8](#_ENREF_8)] |
| 67b | 39.14 | 391 | 164.07,147.04,103.06, 59.02 | 392 | C_17_H_28_O_10_ | Methylated lamiol | - | + | RG | [[8](#_ENREF_8)] |
| 67c | 39.62 | 299 | 239.17,110.98,96.96 | 300 | C_13_H_16_O_8_ | Deglycosylation product of mudanpioside C | - | + | PS | [[2](#_ENREF_2)] |
| 67d | 42.00 | 495 | 326.19,299.13,165.07 | 496 | C_23_H_28_O_12_ | Deglycosylation product of benzoyloxypaeoniflorin | - | + | PL | [[2](#_ENREF_2)] |
| 67e | 46.10 | 643 | 311.21,311.19,214.05,130.08 | 644 | C_32_H_36_O_14_ | Methylated mudanpioside J | - | + | PS | [[2](#_ENREF_2)] |
| 67f | 49.92 | 653 | 337.24,315.25,110.98 | 654 | C_30_H_38_O_16_ | Deglycosylation product of cistanoside A | - | + | RG | [[11](#_ENREF_11)] |
| 67j | 50.98 | 501 | 457.41,304.23,303.23,217.12 | 502 | C_22_H_30_O_13_ | Deglycosylation product of martynoside | - | + | RG | [[18](#_ENREF_18)] |
| 67h | 52.50 | 487 | 443.34 | 488 | C_21_H_28_O_13_ | Deglucuronide product of acteoside | - | + | RG | [[1](#_ENREF_1)] |
| 67i | 53.60 | 623 | 628.48,447.38,277.22,175.03 | 624 | C_26_H_40_O_17_ | Deglucuronide product of Jionoside A1 | - | + | RG | [[1](#_ENREF_1)] |

*Constituents identified on the basis of comparison with standard available in the lab, T_R_(min): Retention time, +: Detected, -: Not detected, XDT: Xijiao Dihuang Tang, BB: *Bubalus bubalis* Linnaeus, RG: *Rehmannia glutinosa* Libosch., PL: *Paeonia lactiflora* Pall., PS: *Paeonia suffruticosa* Andr.

**Supplementary Table 1B (Tab. S1B ) Identification results of constituents of extract and serum samples of XDT in positive mode by HPLC-Q-TOF-MS.**

| No | T_R_(min) | ESI^+^m/z | | MW | MF | Name | Detected | | Source | Reference |
| --- | --- | --- | --- | --- | --- | --- | --- | --- | --- | --- |
|  |  | MS | MS/MS |  |  |  | Serum | Vitro |  |  |
| 3 | 11.52 | 134 | 88.04,74.03,70.03 | 133 | C_4_H_7_NO_4_ | Aspartic Acid | + | + | BB | [[19](#_ENREF_19)] |
| 4 | 12.47 | 116 | 70.07 | 115 | C_5_H_9_NO_2_ | Proline | + | + | BB | [[19](#_ENREF_19)] |
| 7 | 12.92 | 175 | 130.10,70.07,60.06 | 174 | C_6_H_14_N_4_O_2_ | Arginine | + | + | BB | [[19](#_ENREF_19)] |
| 56* | 21.50 | 268 | 136.06,119.04 | 267 | C_10_H_13_N_5_O_4_ | Adenosine | + | + | BB | - |
| 67 | 35.67 | 126 | 126.06, 108.04 | 125 | C_2_H_7_NO_3_S | Taurine | + | + | BB | [[20](#_ENREF_20)] |

*Constituents identified on the basis of comparison with standard available in the lab, T_R_(min): Retention time, +: Detected, -: Not detected. , XDT: Xijiao Dihuang Tang, BB: *Bubalus bubalis* Linnaeus

**References**

1. B. Zhang, Z. Jiang, Y. Wang, L. Yang, F. Yang, and H. Yu. "Analysis of chemical constituents in fresh,dried and prepared Rehmanniae Radix by UPLC/ESI-Q-TOF MS," *Chinese Traditional Patent Medicine, vol.* 38, no. 5, pp. 1104-1108.

2. J. He, Y. Dong, X. Liu, et al. "Paeonia suffruticosaComparison of Chemical Compositions, Antioxidant, and Anti-Photoaging Activities of Flowers at Different Flowering Stages," *Antioxidants (Basel, Switzerland), vol.* 8, no. 9.

3. X. Nan, S. Su, K. Ma, et al. "Bioactive fraction of Rhodiola algida against chronic hypoxia-induced pulmonary arterial hypertension and its anti-proliferation mechanism in rats," *Journal of Ethnopharmacology, vol.* 216, pp. 175-183.

4. F.I. Abu Bakar, M.F. Abu Bakar, N. Abdullah, S. Endrini, and S. Fatmawati. "Optimization of Extraction Conditions of Phytochemical Compounds and Anti-Gout Activity of Euphorbia hirta L. (Ara Tanah) Using Response Surface Methodology and Liquid Chromatography-Mass Spectrometry (LC-MS) Analysis," *Evidence-Based Complementary and Alternative Medicine, vol.* 2020, p. 4501261.

5. C. Xiao, M. Wu, Y. Chen, Y. Zhang, X. Zhao, and X. Zheng. "Revealing metabolomic variations in Cortex Moutan from different root parts using HPLC-MS method," *Phytochemical analysis : PCA, vol.* 26, no. 1, pp. 86-93.

6. P. Jiang, Y. Ma, Y. Gao, et al. "Comprehensive Evaluation of the Metabolism of Genipin-1-β-d-gentiobioside in Vitro and in Vivo by Using HPLC-Q-TOF," *Journal of Agricultural and Food Chemistry, vol.* 64, no. 27, pp. 5490-5498.

7. S.L. Li, J.Z. Song, F.F. Choi, et al. "Chemical profiling of Radix Paeoniae evaluated by ultra-performance liquid chromatography/photo-diode-array/quadrupole time-of-flight mass spectrometry," *Journal of Pharmaceutical and Biomedical Analysis, vol.* 49, no. 2, pp. 253-266.

8. Q. Song, Y. Zhao, N. Zhang, et al. "Establishment of HPLC fingerprint of Rehmanniae Radix and its HPLC-ESI-MS analysis," *Chinese Traditional and Herbal Drugs, vol.* 47, no. 23, pp. 4247-4252.

9. L. Zhu, S. Sun, Y. Hu, and Y. Liu. "Metabolic study of paeoniflorin and total paeony glucosides from Paeoniae Radix Rubra in rats by high-performance liquid chromatography coupled with sequential mass spectrometry," *Biomedical Chromatography, vol.* 32, no. 4.

10. J. Liu, L. Chen, C.-r. Fan, et al. "Qualitative and quantitative analysis of major constituents of Paeoniae Radix Alba and Paeoniae Radix Rubra by HPLC-DAD-Q-TOF-MS/MS," *Zhongguo Zhong yao za zhi = Zhongguo zhongyao zazhi = China journal of Chinese materia medica, vol.* 40, no. 9, pp. 1762-1770.

11. L. Li, R. Tsao, R. Yang, C. Liu, J.C. Young, and H. Zhu. "Isolation and purification of phenylethanoid glycosides from Cistanche deserticola by high-speed counter-current chromatography," *Food Chemistry, vol.* 108, no. 2, pp. 702-710.

12. Y.H. Shi, S. Zhu, Y.W. Ge, et al. "Characterization and quantification of monoterpenoids in different types of peony root and the related Paeonia species by liquid chromatography coupled with ion trap and time-of-flight mass spectrometry," *Journal of Pharmaceutical and Biomedical Analysis, vol.* 129, pp. 581-592.

13. R.H. Zhang, C.R. Li, H. Yang, et al. "An UPLC-MS/MS method for simultaneous determination of multiple constituents in Guizhi Fuling capsule with ultrafast positive/negative ionization switching," *Chinese Journal of Natural Medicines, vol.* 16, no. 4, pp. 313-320.

14. D. Rocha-Parra, J. Chirife, C. Zamora, and S. de Pascual-Teresa. "Chemical Characterization of an Encapsulated Red Wine Powder and Its Effects on Neuronal Cells," *Molecules (Basel, Switzerland), vol.* 23, no. 4.

15. W. ZHAO, Y. LIN, P. LI, and Y. LIU. "Analysis of chemical constituents of Moutan cortex by HPLC-QTOFMS," *Journal of Pharmaceutical Practice, vol.* 32, no. 04, pp. 261-265.

16. N. Penner, R. Ramanathan, J. Zgoda-Pols, and S. Chowdhury. "Quantitative determination of hippuric and benzoic acids in urine by LC-MS/MS using surrogate standards," *Journal of Pharmaceutical and Biomedical Analysis, vol.* 52, no. 4, pp. 534-543.

17. I. Rjeibi, A. Ben Saad, S. Ncib, S. Souid, M. Allagui, and N. Hfaiedh. "Brachychiton populneus as a novel source of bioactive ingredients with therapeutic effects: antioxidant, enzyme inhibitory, anti-inflammatory properties and LC-ESI-MS profile," *Inflammopharmacology, vol.* 28, no. 2, pp. 563-574.

18. H. Kirmizibekmez, P. Montoro, S. Piacente, C. Pizza, A. Donmez, and I. Calis. "Identification by HPLC-PAD-MS and quantification by HPLC-PAD of phenylethanoid glycosides of five Phlomis species," *Phytochemical Analysis, vol.* 16, no. 1, pp. 1-6.

19. W. van Helmond and M. de Puit. "Amino Acid Profiling from Fingerprints Using Amide Stationary-Phase UPLC-MS," *Methods in molecular biology (Clifton, NJ), vol.* 2030, pp. 439-450.

20. S. Guiraud, I. Montoliu, L. Da Silva, et al. "High-throughput and simultaneous quantitative analysis of homocysteine-methionine cycle metabolites and co-factors in blood plasma and cerebrospinal fluid by isotope dilution LC-MS/MS," *Analytical and Bioanalytical Chemistry, vol.* 409, no. 1, pp. 295-305.
