# Supplementary material for: Serum Pharmacochemistry Combining Network Pharmacology to Discover the Active Constituents and Effect of Xijiao Dihuang Tang Prescription for Treatment of Blood-Heat and Blood-Stasis Syndrome-Related Disease
Source: Oxid Med Cell Longev. 2022 Feb 7;2022:6934812. doi: 10.1155/2022/6934812 (PMC8845118; doi:10.1155/2022/6934812)
Supplement: Supplementary Materials — Table S1: HPLC-QTOF/MS/MS analysis of SCXDT. Table S2: compound targets for each serum compound of XDT. [file 6934812.f1.zip › table s2.docx]

**Supplementary Table 2 (Tab. S2) Potential Targets of serum constituents in Xijiao Dihuang Tang (XDT)**

| **NO** | **Compounds** | **Targets** |
| --- | --- | --- |
| 1 | Guanosine | ADORA1, ADORA2A, ADORA3, HSPA8, HSPA5, MCL1, ADK, DPP4, ADA, EIF4E, PNP, GAPDH, HPRT1, AHCY, CDC42, RAC1, POLA1, CDA, TYMP, CD38, CA2, CA1, CA12, CA9, GBA, GAA, MAPK1, OGA, TERT, CCND1, FUCA1, TK1, GRK1, EHMT1, EHMT2, CA7, CA13, CA14, DAO, EGFR, SETD7, TYR, F2, SRM, HK2, HK1, PDCD4, CDK4 |
| 2 | Aspartic Acid | NOS1, ABAT, GOT1, GABRA1, CTSD, CDC25B, BCHE, ADH1C, GLUD1, GOT2, CKM, TPI1, ME2, ALOX5, GRIA1, PYGM, OTC, AGXT, P3H3, GATM, ADSS1, ME3, AAcubin, SLC25A12, SDHA, MAT2A, GLUD2, GRIK2, SLC25A13, EGLN1 |
| 3 | Proline | GABRA3, GABRA2, SLC6A1, ACE, REN, GABRR1, ANPEP, EGLN1, GABBR2, F2, LTA4H, GABRB2, GAB8-Epiloganic acid, GABBR1, GABRA1, CTSD, PPIA, BCHE, NOS3, DAO, GATM, DPP4, DPEP1, GABRA6, PTGS2, ADH1A |
| 4 | Arginine | NOS2, NOS1, AAucubin, CA2, CA1, CA12, CA9 |
| 5 | Adenosine | ADORA1, ADORA2A, ADORA3, DPP4, ADK, HSPA8, HSPA5, ADA, AHCY, GAPDH, EHMT1, EHMT2, MCL1, SETD7, EGFR, AMD1, SRM, PDCD4, SRC, ADORA2B, MAPK1, SLC29A1, PNP, SETD2, CARM1, PRMT1, DOT1L, GRK1, P2RY1, P2RY11, MTAP, ROCK2, PRKACA, FBP1, SMS, CDA, FHIT, QARS, KMT2A, SUV39H1, DNMT1, INMT, SMYD2, EZH2, EZH1, SETDB1, PNMT, DNMT3B, MAPKAPK2, HSPA1A, CA2, CA1, CA12, CA9, RARS, OGA, CCND1, GSK3B, GBA, GAA, PIM1, FUCA1, CA14, HSD17B1, MARS, IDO1, PRMT7, PARG, F2, SLC28A2, IARS, DAO, CDK4, PTGS1, PTGS2 |
| 6 | Taurine | GATM, ME2 |
| 7 | Aucubin | PTGS2, CA2, DPP4, RELA, BCL2, BAX, TNF, IL6, NFKBIA, TREH, ADORA1, ADORA2A, FUCA1, CDA, FOLH1, HK2, HK1, LGALS3, LGALS9, CA1, CA12, CA14, CA9, HPRT1, ADA, TYR |
| 8 | 8-Epiloganic acid | CA2, DPP4, PTGS2, ENGASE, NEU3, NEU2, NEU4, SELP, ADORA1, CA1, CA12, CA9, OGA, MGAM, TREH, NAALAD2, SELL |
| 9 | Catalpol | DPP4, BCL2, CASP3, SOD1, TREH, CA2, FUCA1, CA1, CA12, CA9, AMY2A, FOLH1, ADA, ADORA1, ADORA2A, CA14, AKR1B1, HPRT1, HK2, HK1 |
| 10 | Geniposidic acid | CA2, ADORA1, SELP, ENGASE, MGAM, YARS |
| 11 | Salidroside | TYR, CA2, CA7, CA1, CA3, CA6, CA12, CA9, CA4, CA5A, CA14, ADA, ADK, ADORA2A, GSK3B, CDA, AKR1C3, TYMP, HRAS, PNP, GBA, P2RX3, ADORA3 |
| 12 | Geniposide | GABRA2, BCL2, HMOX1, GAP43, PLB1, NOS3, GSTM2, ADORA1, ADORA2A, CA2, CA1, CA12, CA9, ADORA3, LGALS3, LGALS9, CA14, EPHX2, SLC5A2, SLC29A1, ADORA2B, FUCA1, TYR, ADK, SLC5A4, SLC5A1, ATIC, ALOX12, CA7, CA4, CA13, CA5A, HRAS, LGALS4, AKR1B1, TYMP, LGALS8, CA6, ADA, EIF4H, PABPC1, MMP2, HSP90AA1, AKR1C3, PYGL, MMP13, MMP1, MMP7, PNP, MMP12, MMP8, GBA, OGA, HK2, HK1, SLC28A3 |
| 13 | Rehmapicroside | PPP1CC, PPP2CA, PPP2R5A, HSD11B2, HSD11B1, PTPN1, GLRA1, GLRA2, STAT3, SELP, RORC, ATP1A1, BCL2L1, PLA2G1B, PRKCA, SELL, ENGASE, PTAFR, PRKCE, PRKCH, PRKCQ, CASP3, CASP6, CASP7, CASP8, CASP1, KDR, MME |
| 14 | Vanillic acid | PTGS2, MAOB, MAOA, ADRA2A, PTGS1, NOS3, TYRP1 |
| 15 | 8-Debenzoylpaeoniflorin | CDK1, VEGFA, FGF1, FGF2, HPSE, HSP90AA1, PSEN2, LGALS4, LGALS3, LGALS8, HTR2B, ADRA2A, ADRA2C, ADRA2B, DRD1, DRD2, ADRA1D, HTR2A, HTR2C, DRD3, CYP2D6, HTR6, ADRA1A, HTR1B, RORC, TRPV1, STAT3, PSENEN, NCSTN, APH1A, PSEN1, APH1A, PSEN1, APH1B |
| 16 | 1-O-β-D-glucopyransoyl-paeonisuffron | HTR2B, ADRA2A, ADRA2C, ADRA2B, DRD1, DRD2, ADRA1D, HTR2A, HTR2C, DRD3, CYP2D6, HTR6, ADRA1A, HTR1B, RORC, PPM1A, STAT3, VEGFA, FGF1, FGF2, HPSE, FDFT1, GLRA1, GLRA2, PTAFR, CDK1, OPRK1, MLNR, HSP90AA1, VDR, LGALS4, LGALS3, LGALS8, KCNH2, PSEN2, PSENEN, NCSTN, APH1A, PSEN1, APH1B |
| 17 | Mudanpioside F | PTPN1, PPM1B, PPP1CC, PPP2CA, PPP2R5A, ADA, CDA, HSD11B2, STAT3, TYR, FUCA1, RORC, ATP1A1, OGA, CDK2, PRKCA, AKR1C3, PIN1, ADORA2A, AHCY, BCL2L1, CDK9, CSNK2A1, HDAC1, GBA, ADK, PNP, FOLH1, DAO, HSPA8, TK1, GAPDH, F2, FBP1, PTAFR, MMP3, MMP1, ADAM17, KDM4C, GLRA1, GLRA2, HSPA5, SLC5A2, TYMS, MAPK1, AKR1C2, HSD11B1, GRK1, IARS, CCNA1, CCNA2, CCNT1 |
| 18 | Oxypaeoniflorin | HSP90AA1, LGALS3, LGALS9, F10, AMY1A, PTPN1, SERPINE1, PTPN2, BACE1, SQLE, AKR1B1, SLC6A2, SSTR5, SSTR2, SSTR4, SSTR1, SSTR3, ABCB1, VEGFA, FGF1, FGF2, HPSE, TDP1 |
| 19 | Paeoniflorin | TNF, IL6, CD14, LBP, LGALS3, LGALS9, HSP90AA1, SLC6A2, SSTR5, SSTR2, SSTR4, SSTR1, SSTR3, ABCB1, VEGFA, FGF1, FGF2, HPSE, F10, AMY1A, PTPN1, SERPINEl, PTPN2, BACE1, SQLE, PTAFR, SELP, AKR1B1 |
| 20 | Mudanoside A | PTGS2, ERN1, HMGCR, EP300, CA9, CA2, CA1, ALPG, PLAA, MAOB, CA7, CA12, CA14, FASN, KCNMA1, CISD1, PIM3, CDC25B, MB, ACHE, NAT1, GSK3B, MAOA, ASF1A, PLEC, CSNK1Al, CSNK1D, PTPN22 |
| 21 | Paeonol | PTGS1, ADRB1, PTGS2, ADRA2A, ADRA2C, SLC6A2, ADRA2B, ADRA1B, SLC6A3, ADRB2, ADRA1D, MAOB, MAOA, CTRB1, ADRA1A, CHRM1, CHRM2, RELA, AKT1, BCL2, BAX, MAPK1, TNF, AHSA1, NFKBIA, ICAM1, IL2, TYR |
| 22 | Mudanpioside D | HSP90AA1, LGALS3, LGALS9, AMY1A, SERPINE1, CA14, ADORA2A, ADORA3, MGMT, ADORA2B, AKR1B1, BACE1, TDPl, ABL1, EPHA2, LCK, SRC, KDR, MAP3K9, FGFR1, AURKA, BTK, SLC5A2, VARS, LARS, IGFBP3, ADK, MMP13, MMP1, MMP7, MMP8, PTPN1, ADORAl, F10, SSTR2, SSTR4, SSTR1, SSTR3, LGALS7, IMPDH1, VEGFA, FGF1, FGF2, HPSE, SQLE |
| 23 | Methyl vanillate | CA2, CA7, CA1, CA12, CA14, CA9, CA6, FUT7, CA4, CA5A, POLA1, POLB, CA3, SERPINE1, ERN1, SQLE, CA13, MAOA, AKR1C3, CA5B, FYN, MAOB, EGLN1, ALOX15, CDK5R1, CDK2, CDK9, DYRK1, PLEC, CSNK1A1, CSNK1D, TYMS, TPMT, MB, TERT, CHRM1, GSTA1, TTR, CDK5, CCNA1, CCNT1, CCNA2 |
| 24 | Rehmannioside D sulfate | MAPK11, MAPK14, TNF, IL1B, IL6 |
| 25 | Lamiol sulfate | MAPK11, MAPK14, TNF, IL1B, IL6 |
| 26 | Jionoside A1 sulfate | MAP2K1, MAP2K2, MAPK11, MAPK14, PTGS2, TNF, IL1B, IL6 |
| 27 | Deglycosylation product of dihydrocatalpol | MAPK11, MAPK14, TNF, IL1B, IL6 |
| 28 | Deglucose product of lamiol | MAPK11, MAPK14, TNF, IL1B, IL6 |
| 29 | Deglucuronide product of 6-O-E-Feruloylajugol | MAP2K1, MAP2K2, MAPK11, MAPK14, PTGS2, TNF, IL1B, IL6 |
| 30 | Deglycosylation product of cistanoside F | MAP2K1, MAP2K2, MAPK11, MAPK14, PTGS2, TNF, IL1B, IL6 |
| 31 | Methylated cistanoside F | MAP2K1, MAP2K2, MAPK11, MAPK14, PTGS2, TNF, IL1B, IL6 |
| 32 | Methylated lamiol | MAPK11, MAPK14, TNF, IL1B, IL6 |
| 33 | Deglucuronide product of Jionoside A1 | MAP2K1, MAP2K2, MAPK11, MAPK14, PTGS2, TNF, IL1B, IL6 |
| 34 | Deglycosylation product of cistanoside A | MAP2K1, MAP2K2, MAPK11, MAPK14, PTGS2, TNF, IL1B, IL6 |
| 35 | Deglycosylation product of martynoside | MAP2K1, MAP2K2, MAPK11, MAPK14, PTGS2, TNF, IL1B, IL6 |
| 36 | Deglucuronide product of acteoside | MAP2K1, MAP2K2, MAPK11, MAPK14, PTGS2, TNF, IL1B, IL6 |
| 37 | Galloyl paeoniflorin sulfate | MAP3K7, MAP4K2, MAP2K1, MAP2K2, MAPK11, MAPK14, TNF, IL1B, IL6 |
| 38 | Galloyl paeoniflorin glucuronide | MAP3K7, MAP4K2, MAP2K1, MAP2K2, MAPK11, MAPK14, TNF, IL1B, IL6 |
| 39 | Benzoyloxypaeoniflorin sulfate | MAP3K7, MAP4K2, MAP2K1, MAP2K2, MAPK11, MAPK14, TNF, IL1B, IL6 |
| 40 | 1,2,3,6-Tetragalloyl glucose sulfate | MAP2K1, MAP4K2, MAP2K2, MAPK11, MAPK14, PTGS2, TNF, IL1B, IL6 |
| 41 | Mudanpioside E glucuronide | MAP3K7, MAP4K2, MAP2K1, MAP2K2, MAPK11, MAPK14, PTGS2, TNF, IL1B, IL6 |
| 42 | Oxidation albiflorin | MAP3K7, MAP4K2, MAP2K1, MAP2K2, MAPK11, MAPK14, TNF, IL1B, IL6 |
| 43 | Oxymudanpioside I | MAP3K7, MAP4K2, MAP2K1, MAP2K2, MAPK11, MAPK14, TNF, IL1B, IL6 |
| 44 | Deglycosylation product of mudanpioside I | MAP3K7, MAP4K2, MAP2K1, MAP2K2, MAPK11, MAPK14, TNF, IL1B, IL6 |
| 45 | Methylated mudanpioside E | MAP3K7, MAP4K2, MAP2K1, MAP2K2, MAPK11, MAPK14, PTGS2, TNF, IL1B, IL6 |
| 46 | Deglycosylation product of 1,2,3,4,6-Pentagalloyl glucose | MAP2K1, MAP2K2, MAPK11, MAPK14, PTGS2, TNF, IL1B, IL6 |
| 47 | Deglycosylation product of benzoyloxypaeoniflorin | MAP3K7, MAP4K2, MAP2K1, MAP2K2, MAPK11, MAPK14, TNF, IL1B, IL6 |
| 48 | Quercetin 3'-sulfate | MAP2K1, MAP2K2, MAPK11, MAPK14, PTGS2, TNF, IL1B, IL6 |
| 49 | Syringic acid sulfate | MAP2K1, MAP2K2, MAPK11, MAPK14, PTGS2, TNF, IL1B, IL6 |
| 50 | Mudanoside B sulfate | MAP2K1, MAP2K2, MAPK11, MAPK14, PTGS2, TNF, IL1B, IL6 |
| 51 | Genipin sulfate | MAP2K1, MAP2K2, MAPK11, MAPK14, PTGS2, TNF, IL1B, IL6 |
| 52 | Syringic acid glucuronide | MAP2K1, MAP2K2, MAPK11, MAPK14, PTGS2, TNF, IL1B, IL6 |
| 53 | 1,6-Bis-O-galloyl-beta-D-glucose | MAP2K1, MAP2K2, MAPK11, MAPK14, PTGS2, TNF, IL1B, IL6 |
| 54 | Deglycosylation product of suffruticoside A | MAP2K1, MAP2K2, MAPK11, MAPK14, PTGS2, TNF, IL1B, IL6 |
| 55 | Deglycosylation product of suffruticoside D | MAP2K1, MAP2K2, MAPK11, MAPK14, PTGS2, TNF, IL1B, IL6 |
| 56 | Deglycosylation product of suffruticoside B | MAP2K1, MAP2K2, MAPK11, MAPK14, PTGS2, TNF, IL1B, IL6 |
| 57 | Methylated genipin | MAP2K1, MAP2K2, MAPK11, MAPK14, PTGS2, TNF, IL1B, IL6 |
| 58 | Deglycosylation product of suffruticoside C | MAP2K1, MAP2K2, MAPK11, MAPK14, PTGS2, TNF, IL1B, IL6 |
| 59 | Deglycosylation product of mudanpioside C | MAP3K7, MAP4K2, MAP2K1, MAP2K2, MAPK11, MAPK14, TNF, IL1B, IL6 |
| 60 | Methylated mudanpioside J | MAP3K7, MAP4K2, MAP2K1, MAP2K2, MAPK11, MAPK14, PTGS2, TNF, IL1B, IL6 |
